# Tracking pan-continental trends in environmental contamination using sentinel raptors—what types of samples should we use?

**DOI:** 10.1007/s10646-016-1636-8

**Published:** 2016-03-05

**Authors:** S. Espín, A. J. García-Fernández, D. Herzke, R. F. Shore, B. van Hattum, E. Martínez-López, M. Coeurdassier, I. Eulaers, C. Fritsch, P. Gómez-Ramírez, V. L. B. Jaspers, O. Krone, G. Duke, B. Helander, R. Mateo, P. Movalli, C. Sonne, N. W. van den Brink

**Affiliations:** Department of Toxicology, Faculty of Veterinary Medicine, University of Murcia, Campus de Espinardo, 30100 Murcia, Spain; Section of Ecology, Department of Biology, University of Turku, 20014 Turku, Finland; FRAM—High North Research Centre for Climate and the Environment, Norwegian Institute for Air Research, 9296 Tromsø, Norway; NERC Centre for Ecology and Hydrology, Lancaster Environment Centre, Library Avenue, Bailrigg, Lancaster, LA1 4AP UK; Institute for Environmental Studies, VU University, De Boelelaan 1087, 1081 HV Amsterdam, The Netherlands; Chrono-Environnement, UMR 6249 University Bourgogne Franche-Comté/CNRS Usc INRA, 16 Route de Gray, 25030, Besançon Cedex France; Behavioural Ecology and Ecophysiology Group, Department of Biology, University of Antwerp, Universiteitsplein 1, 2610 Wilrijk, Belgium; Department of Biology, Norwegian University of Science and Technology, EU2-169, Høgskoleringen 5, 7491 Trondheim, Norway; Leibniz Institute for Zoo and Wildlife Research, Alfred-Kowalke-Strasse 17, 10315 Berlin, Germany; Centre for the Environment, Oxford University Environmental Change Institute, South Parks Road, Oxford, OX1 3QY UK; Environmental Research & Monitoring, Swedish Museum of Natural History, Box 50007, SE-104 05 Stockholm, Sweden; Instituto de Investigación en Recursos Cinegéticos-IREC (CSIC-UCLM-JCCM), Ronda de Toledo s/n, 13071 Ciudad Real, Spain; Department of Collections, Naturalis Biodiversity Center, Darwinweg 2, 2333 CR Leiden, The Netherlands; Department of Bioscience, Artic Research Centre (ARC), Århus University, Frederiksborgvej 399, PO Box 358, 4000 Roskilde, Denmark; Division of Toxicology, Wageningen University, PO Box 8000, NL-6700EA Wageningen, The Netherlands; Deltares, Marine and Coastal Systems, P.O. Box 177, 2600 MH Delft, The Netherlands

**Keywords:** Bird of prey, Contaminant, Monitoring, Sample type, Matrix

## Abstract

Biomonitoring using birds of prey as sentinel species has been mooted as a way to evaluate the success of European Union directives that are designed to protect people and the environment across Europe from industrial contaminants and pesticides. No such pan-European evaluation currently exists. Coordination of such large scale monitoring would require harmonisation across multiple countries of the types of samples collected and analysed-matrices vary in the ease with which they can be collected and the information they provide. We report the first ever pan-European assessment of which raptor samples are collected across Europe and review their suitability for biomonitoring. Currently, some 182 monitoring programmes across 33 European countries collect a variety of raptor samples, and we discuss the relative merits of each for monitoring current priority and emerging compounds. Of the matrices collected, blood and liver are used most extensively for quantifying trends in recent and longer-term contaminant exposure, respectively. These matrices are potentially the most effective for pan-European biomonitoring but are not so widely and frequently collected as others. We found that failed eggs and feathers are the most widely collected samples. Because of this ubiquity, they may provide the best opportunities for widescale biomonitoring, although neither is suitable for all compounds. We advocate piloting pan-European monitoring of selected priority compounds using these matrices and developing read-across approaches to accommodate any effects that trophic pathway and species differences in accumulation may have on our ability to track environmental trends in contaminants.

**Electronic supplementary material:**

The online version of this article (doi:10.1007/s10646-016-1636-8) contains supplementary material, which is available to authorized users.

## Introduction

Raptors (birds of prey and owls) were among the first wildlife species known to be affected by anthropogenic pollutants. Pesticide and/or contaminant related declines in peregrine falcon (*Falco peregrinus*), bald eagle (*Haliaeetus leucocephalus*), white-tailed sea eagle (*Haliaeetus albicilla*) and sparrowhawk (*Accipiter nisu*s) populations have been widely documented in Europe and North America in the 20th Century (Hickey 1969; Ratcliffe 1980; Nisbet 1989; Newton and Wyllie 1992; Rutz et al. 2006; Sielicki and Mizera 2009). These were largely caused by primary and/or secondary exposure to organochlorine pesticides (OCPs), such as dichlorodiphenyltrichloroethanes (DDTs) and cyclodiene pesticides (Drins), other organochlorine pollutants such as polychlorinated biphenyls (PCBs), and toxic metals such as mercury (Hg) and lead (Pb) (Ratcliffe 1970; García-Fernández et al. 1995, 1996, 2005a, b; Weech et al. 2003; Mateo 2009). Organochlorine pesticides and pollutants (OCs) are now restricted in their use or banned in many countries but they are highly persistent in the environment and lower level exposure still occurs, particularly in higher trophic level wildlife (Sonne et al. 2010; Eulaers et al. 2011b; Luzardo et al. 2014). Other threats remain. For example, Pb intoxication in birds of prey, caused by ingestion of Pb shot and ammunition fragments in unretrieved game, is an ongoing concern for raptor populations (Krone et al. 2004, 2006, 2009; García-Fernández et al. 2005a; Hunt et al. 2006; Helander et al. 2009; Finkelstein et al. 2012; Group of Scientists 2014). There is also exposure to newer compounds, such as new brominated and organophosphate flame retardants and pharmaceuticals (Cuthbert et al. 2011; Covaci et al. 2011). Many of these substances are associated with adverse health effects on the endocrine, immune, nervous and reproductive systems in both humans (Lutz et al. 1999; Yorifuji et al. 2008; Hatcher-Martin et al. 2012) and wildlife (Mateo et al. 2003; Cortinovis et al. 2008; Henny et al., 2009; Naidoo et al. 2009; Frederick and Jayasena 2010; Harris and Elliott 2011). In some cases, they can cause widespread acute mortality and endanger the survival of species; examples include the effects of diclofenac on *Gyps* vultures in Asia (Oaks et al. 2004) and Pb in the California condor (*Gymnogyps californianus*; Finkelstein et al. 2012).

The vulnerability of raptors to environmental contaminants is in part due to their trophic position at the top of foodpyramids. Persistent, bioaccumulative contaminants can be transferred, and in some cases biomagnified, along food chains and accumulated in high concentrations by apex predators (Furness 1993; Elliott et al. 2009; Guigueno et al. 2012). In addition, raptors are vulnerable to (primary or secondary) poisoning because of facultative scavenging and of an increased likelihood that sick and moribund prey are likely to be caught (Langlier 1993; Green et al. 2004), as has been demonstrated for strychnine (Martínez-López et al. 2006) and is likely to occur for currently-used compounds such as anticoagulant rodenticides and anticholinesterase compounds (e.g. Rattner et al. 2014a). Early observations on the impacts of OCs on raptors spawned an increase in the numbers of analytical studies using raptor tissues and eggs and subsequent recognition that raptors can be powerful sentinels of marine and terrestrial environmental contamination (Sergio et al. 2005; Rattner 2009). As a result, raptors have been widely used in some regions of the world in biomonitoring programs. Such programmes assess spatial and temporal trends in concentrations of bioaccumulative environmental chemicals and investigate associated effects on populations (Gómez-Ramírez et al. 2014). They can provide early warning of the potential impacts in humans (Pain et al. 2010), protected wildlife species and on the wider environment and they can be used to track the success of mitigation in reducing exposure (Helander 2003; Shore et al. 2005; García-Fernández et al. 2005b).

In the European Union (EU), regulation of industrial chemicals, plant protection products, pharmaceuticals, and biocides is governed by directives such as Regulation (EC) No 1907/2006 and ammendements (REACH—Registration, Evaluation, Authorisation & Restriction of Chemicals), Regulation (EC) No 1107/2009 concerning the authorisation of plant protection products, Regulation (EC) No 726/2004 concerning the authorisation of human and verterinary pharmaceuticals, and The Biocidal Product Regulation (BPR, Regulation (EU) 528/2012). These directives apply across all EU Member States but lack any monitoring of their effectiveness in protecting against environmental pollution. This may be partly because such monitoring is challenging given the large spatial scales involved. However, a recent survey (Gómez-Ramírez et al. 2014) identified a number of European long-term, national scale, biomonitoring programmes that use raptors as sentinels of environmental contamination. Although almost all the programmes are restricted to western European countries, the types of biological samples needed for analysis are routinely collected throughout all of Europe, typically as part of surveys of raptor populations (Derlink et al. in preparation). Incorporation of such samples into existing contaminant monitoring programmes could extend the reach of pollution monitoring to a pan-European scale and thereby provide assessment of the effectiveness of regulation of chemicals across the EU.

Coordination and integration of large spatial scale monitoring across multiple independent monitoring programmes is challenging. It requires mutual sharing, dissemination and adoption of best practice in terms of the types of samples collected and analysed (Gómez-Ramírez et al. 2014). Sample matrices vary in the information they provide about exposure and effects and not all are suitable for biomonitoring. The scientific relevance of each sample type for monitoring depends on the objectives of the study, for example if the monitoring program is intended to detect contaminant trends or evaluate effects, and if so at what level of biological organization. Furthermore, choice of matrix should ideally be related to the toxicokinetics and toxicodynamics of the compounds of interest and the site of toxic action (Walker et al. 2008). It is also important to use matrices in which pollutant concentrations are above the analytical limit of detection in a significant proportion of the samples and, more pragmatically, to use those types of samples that are likely to be widely available. However, we are unaware of any published evaluation that examines the potential widescale availability of different types of sample for analysis or that evaluates their usefulness and limitations for monitoring contaminants. This lack of information presents a significant barrier to developing EU-scale monitoring for contaminants using raptor tissues.

Our aim in the present study was to undertake the first ever assessment of the potential widescale availability of raptor samples in Europe for biomonitoring and the relative merits of each. We combined data from a recent European inventory on contaminant monitoring in raptors (Gómez-Ramírez et al. 2014) with a survey of raptor population monitoring activities that are ongoing over the same geographical area (Derlink et al. in preparation). This allowed us to ascertain which sample matrices might be most available for monitoring and which contaminants are currently prioritized for monitoring in birds of prey and owls in Europe. These earlier surveys did not provide information on which sample types are predominantly used to analyse different contaminants. We therefore additionally reviewed 249 papers published between 1966 and 2015, which reported contaminant concentrations in raptors in Europe. This provided information on sample types most widely used to monitor each of our identified priority contaminant classes. Details of the methods by which we searched the literature are given in Supporting Information S.I. Document 1. Our specific objectives were to: (i) determine which raptor samples are currently collected across Europe; (ii) assess which sample types are predominantly used to measure priority compound groups, and (iii) review the suitability of these different sample types for contaminant monitoring in general and for priority compounds specifically.

## Collection of different sample types across Europe

Most raptors are protected species and lethal sampling of individuals is not permissible on legal or ethical grounds. Active monitoring of raptors is limited to (invasive and non-invasive) non-destructive sampling. This includes blood and biopsies taken from live birds, plucked feathers and preen oil, and samples taken without contact with a living bird such as moulted feathers, addled or deserted eggs, regurgitated pellets, excrement, and tissues from carcasses that have been found and collected. Collection of samples can be demanding logistically since nests are often difficult to access and collection usually requires skilled and trained personnel, specialised equipment and legal permits. Therefore, if monitoring is to be conducted across continental and other large spatial scales, it is only likely to be feasible if it utilises samples that are already being collected as part of existing monitoring programmes or after careful prior selection of the suitable sample material and species. Two recent inventories collected by questionnaires (Gómez-Ramírez et al. 2014; Derlink et al. in preparation) provide, for the first time, information on the extent of raptor monitoring and sample collection throughout Europe. We combined the data from these two surveys and found that, within Europe, there was information for 281 different raptor monitoring schemes from across 35 European countries (Table 1; S.I. Fig. 1). Of these, 182 programmes from 33 countries collect raptor samples. The United Kingdom (UK), Sweden and Italy are the countries with the highest number of schemes collecting samples, while there appeared to be no sample collections of raptor material in Luxembourg and Serbia (Table 1). There were no responses to the questionnaires from Greece, Lithuania, Albania, Moldova, Macedonia and Montenegro and so we are unaware of any raptor monitoring schemes in those countries.

**Table 1:** Number of schemes that collect each type of sample in raptors from European countries

	Number	Samples collected							
	Schemes	Feather	Egg	Food remains	Carcass	Internal tissues	Blood	Pellets	Preen oil
Austria	5	3	2	4	2		1	2	
Belarus	1	1	1	1			1	1	
Belgium	5	2	1	1	2	1	1	1	1
Bosnia/herzegovina	3							1	
Bulgaria	1	1	1	1		1	1	1	
Croatia	4			2					
Cyprus	2		1	1	1			1	
Czech Republic	1		1	1			1		
Denmark and Greenland	6	2	2	1		1	2		1
Estonia	7	3	1	2	1		2	2	
Finland	8	4	4	2	3	3		1	
France	11	2	4		5	5	2		1
Georgia	2	1			1				
Germany	7	1	6		1	3	2		
Hungary	10	4	3	7	3	1	1	6	
Iceland	1	1	1	1	1	1	1	1	
Ireland	10	8	3	7	9	4	4	6	
Italy	29	11	5	5	9	10	6	4	
Latvia	4	2	2	3	1		1	1	
Luxembourg	2								
Netherlands	4	1	1				1		1
Norway	5	5	2		2	1	3		2
Poland	1						1		
Portugal	14	7	3	5	4	2	4	7	
Romania	3	1		2			1	2	
Russian Federation	2	1	1	2	1	1	1	2	
Serbia	1								
Slovakia	10	3	4	5	4	3	1	3	
Slovenia	11	2	1	2	3	1	1	3	
Spain	9	7	5		5	5	8	4	
Sweden	30	11	9	4	2	7	2	2	
Switzerland	5	2	1		1	1	1		
Turkey	7	3	1	1	1	1	1	2	
Ukraine	2	1						1	
United Kingdom	58	10	25	21	16	10	3	12	
Total schemes	281	100	91	81	78	62	54	66	6
Total countries	35	28	27	23	23	20	27	23	5

Of the different sample types, feathers and addled/deserted eggs are the most frequently collected matrices across the 182 schemes (55 and 50 % of schemes, respectively; Table 1). Food remains (45 %), regurgitated pellets (36 %), internal tissues (34 %), and blood (30 %) were also commonly collected. The questionnaires indicated that carcasses were collected by 78 schemes and presumably crop and gizzard contents and internal organs are likely to be available from those carcasses that are not severely decomposed; the questionnaire returns on the number of schemes collecting food remains and internal tissues may therefore be an underestimate. Preen oil was collected by only a few (3 %) schemes, perhaps reflecting the fact that is only relatively recently that is has been used for monitoring contaminants in raptors (van Den Brink 1997; Yamashita et al. 2007; Jaspers et al. 2008, 2011; Eulaers et al. 2011b).

## What contaminants are measured in different sample matrices?

The scope of current contaminant monitoring programmes using raptors (Gómez-Ramírez et al. 2014) suggests that, in Europe, the priority compounds of interest remain persistent organic pollutants (POPs). These include organochlorine insecticides (measured in 42 schemes from 12 countries), polychlorinated biphenyls (41 schemes from 13 countries) and brominated flame retardants (21 schemes from 10 countries). Perfluorinated compounds which include perfluorooctanesulfonic acid (PFOS), more recently classed as a POP in the Stockholm Convention, are monitored in fewer (7) schemes (from 5 countries); as are dioxins and furans (4 schemes from 3 countries). Two other compound classes, metals/metalloids and anticoagulant rodenticides, are also widely monitored in raptors across Europe (37 schemes from 13 countries and 14 schemes from 6 countries, respectively; Gómez-Ramírez et al. 2014). Although other various compounds are measured as part of investigations into accidental and deliberate poisoning of raptors in Europe, they are not the focus of the present study (see reviews by Berny 2007; Guitart et al. 2010).

Many of the types of raptor sample that are widely collected throughout Europe (Table 1) can be used to monitor these priority contaminants (Gómez-Ramírez et al. 2014). Chemical analysis provides a quantitative measure of exposure/accumulation while genetic and stable isotope analysis of some matrices (feathers, blood and internal tissues) can sometimes be used to identify sources of exposure (Scheuhammer and Templeton 1998; Podlesak et al. 2005; Elliott et al. 2009; Hobson 2011). Other matrices (blood, internal tissues, faeces) are also useful for measuring effect biomarkers (Martinez-Haro et al. 2011b; Bourgeon et al. 2012; Espín et al. 2015). Our review of 249 papers indicated that eggs and liver are the preferred sample matrices when analysing contaminants and were used in twice as many studies as other matrices (Table 2). Studies that used eggs predominantly focussed on POPs (93 % of 88 studies reporting egg contaminant concentrations) whereas liver was used for analysing all the priority compound groups. Most other internal tissue matrices were analysed for a large variety of compound classes, except for fat (used for POPs and perfluorinated compounds only) and bone (predominantly used when quantifying Pb). Of samples that can be taken from live birds, feathers were used in 6–10 fold more studies that preen oil or regurgitated pellets and were analysed for all priority compound groups other than anticoagulant rodenticides.

**Table 2:** Number of published studies (identified from a literature review—see text for details) that analysed different pollutant groups in various sample types from raptor and owl species from Europe

	No/studies in which sample type analysed	No/studies measuring contaminant in each matrix type					
		POPs	PFASs	Lead	Mercury	Cadmium	Anticoagulant rodenticides
		*n* = 137	*n* = 11	*n* = 71	*n* = 59	*n* = 48	*n* = 20
Eggs	88	82	4	11	13	10	0
Feathers	45	10	4	15	21	11	0
Blood/plasma/serum	42	17	3	21	5	15	1
Liver	98	40	3	33	24	23	18
Kidney	40	11	0	21	19	17	0
Muscle	22	15	1	3	3	3	0
Bone	18	0	0	17	0	8	0
Brain	15	7	0	5	2	4	0
Fat	13	12	1	0	0	0	0
Preen oil	7	5	2	0	0	0	0
Regugitated pellets	4	0	0	3	0	0	1

*n* number of studies reporting concentrations of that compound group

Total number of studies reviewed was 249, see S.I. Table 3 for references. Studies often analysed more than one sample type and multiple contaminant groups

Given the potentially wide but variable availability of raptor samples across Europe, it can be difficult to determine which sample matrices may be the best for widescale monitoring of priority compounds. We therefore critically evaluate the factors that affect the usefulness of each matrix for contaminant monitoring in general and for specific priority compound groups in particular. Consideration of sampling, transport and storage of these matrices is also important when selecting matrices for analysis and these are briefly summarised in S.I. Table 1 but are otherwise outside the scope of this paper; they are covered in more detail by Espín et al. (2014a).

## The relative merits of different sample matrices for contaminant monitoring

### Addled and deserted eggs

For both ethical and legal reasons, the taking of unhatched viable eggs is permitted only in exceptional circumstances and requires specific licensing from national regulatory bodies; it is not considered further in the present paper. In contrast, licensed collection and storage of addled and deserted eggs is relatively easy, as reflected by their widespread collection amongst groups that monitor European raptors (91 schemes from 27 countries—Table 1, S.I. Fig. 1). They are important for and widely used in contaminant monitoring studies (Table 2) in part because many pollutants, particularly organic contaminants, are sequestered in eggs (or sometimes eggshells) during formation of the egg. Development of the chick embryo depends on a first phase that consists of the synthesis of lipids by the maternal liver and transport of these lipids to the ovary for incorporation into the maturing oocyte prior to the laying of the egg (Speake et al. 1998). During this process, maternal lipophilic contaminants may be transported along with lipid reserves into the developing oocyte. Thus, contaminant burdens in eggs are directly related to levels in the adult breeding female (Becker and Sperveslage 1989), and reflect exposure in this precise segment of the population that has similar hormonal status and is generally in a healthy condition (Dell’Omo et al. 2008). This is useful for biomonitoring as it may help reduce intra-specific variability in accumulation, although the derived data is not directly indicative of exposure in males and non-breeders.

The necessary restriction of being able to use only failed/addled or deserted eggs limits the number of samples available for analysis. There are also other disadvantages for biomonitoring. Addled eggs, by definition, are a non-random sample in that they only represent failed breeding outcomes. They therefore have a greater likelihood of containing contaminant concentrations that cause adverse effects on hatchability (Henny and Elliott 2007), although failed eggs can sometimes contained higher concentrations than addled eggs (Jaspers et al. 2005). Furthermore, egg sequence is often unknown but can affect pollutant concentrations (Furness and Camphuysen 1997) as can microbial decomposition, the extent of which will depend on the time elapsed between embryo death and collection of the egg (Mulhern and Reichel 1971; Herzke et al. 2002). Biases associated with decomposition are likely to be less significant for highly persistent compound groups but any detection of less persistent transformation products, such as heptachlor *exo*-epoxide and oxychlordane, may be the result of microbiological degradation in the egg and not reflect either maternal exposure or that of successfully hatched young chicks (Herzke et al. 2002).

While egg contaminant concentrations provide information on exposure in breeding females, the breeding strategy of the species can influence whether egg concentrations reflect current local exposure or exposure in other areas used by females before nesting (Nisbet and Reynolds 1984; Henny and Blus 1986). Income breeders fuel reproductive expenditure from food ingested immediately before and during egg production whereas capital breeders utilise stored energy to a much greater extent (Bonnet et al. 1998). Capital breeding females may thus accumulate lipophilic contaminants in lipid laid down while on wintering areas and migration and subsequently transfer those contaminants into eggs on the breeding area. However, raptors are often classified as income breeders as they are largely thought to rely on food intake rather than reserves to service the energetic costs of breeding (Durant et al. 2000), and so egg concentrations in raptor eggs most likely reflect exposure within breeding territories.

Eggs are particularly useful in ecotoxicological studies as contaminant concentrations indicate the load that the nestling is exposed to during embryonic development (García-Fernández et al. 2008). Analysis of egg contents has been widely used to relate exposure to likely or observed reproductive effects (Helander et al. 2002; Lindberg et al. 2004; Herzke et al. 2005; Martinez-Lopez et al. 2007; Vetter et al. 2008; Henny et al., 2009; Pereira et al. 2009; Best et al. 2010; Harris and Elliott 2011; Crosse et al. 2012) and egg concentrations associated with adverse effects on hatching and chick development have been suggested for various contaminants (Blus 2011; Elliott and Bishop 2011; Harris and Elliott 2011; Shore et al. 2011). In addition, eggshell morphometrics can be used to detect shell thinning, an early warning biomarker that occurs at exposures far below those that cause direct impacts on reproduction (Lundholm 1997; Helander et al. 2002); methods to calculate egg weight, length and width, and eggshell weight are described by Espín et al. (2014a) and the relative merits of different ways of calculating shell thickness have been extensively explored (S.I. Document 2). However, it has been argued that, while monitoring of addled eggs may be adequate for evaluating spatial and temporal patterns in contamination, random collection of fresh eggs is needed to best evaluate how reproductive success in the clutch is related to contaminant concentrations (Henny and Elliott, 2007). Such egg collections may be possible for common species which lay two or more eggs, if done over limited spatial scales and at intervals of two or more years and without causing any significant conservation effect. The resultant analysis can provide key information on the ecological importance of the contamination patterns that are revealed through widespread biomonitoring of addled eggs.

Typically, whole egg contents are used for contaminant analysis since it is not possible to separate yolk and albumin in addled eggs. The contents of such eggs can range from undifferentiated material to fully developed embryonic tissue (Espín et al. 2014a). Embryo development can deplete the lipid content of the egg contents and elevate associated concentrations of contaminants expressed on a lipid weight basis (Newton and Bogan 1978; Peakall and Gilman 1979; Helander et al. 1982) and contaminant concentrations in eggs with different stages of embryo development may need to be reported separately. Eggs also lose weight post-laying through diffusive loss of water vapour (Hoyt 1979) and failed eggs can desiccate to varying degrees, although this loss can be estimated from the dimensions of the egg and a species-specific or generalised weight coefficient (Stickel et al. 1973; Best et al., 2010) (S.I. Document 3). Metal and organic contaminant concentrations in eggs are typically expressed on a dry weight and wet or lipid weight basis, respectively and so may need to be corrected and/or normalized for lipid and water content (Espín et al. 2014a); both factors ideally should be reported alongside any concentration data.

In terms of the value of eggs for monitoring particular contaminant groups, we are unaware of any studies that have determined anticoagulant rodenticide residues in raptor eggs (Table 2) although maternal transfer to eggs has been reported in chickens dosed with brodifacoum (Fisher 2009). In contrast, POPs are frequently measured in failed raptor eggs; 60 % of the 137 studies on POPs that we reviewed reported concentrations in eggs (Table 2). These studies largely focussed on legacy POPs but eggs can be used to monitor newer POPs such as poly- and perfluoroalkyl substances (PFASs). A 35-year study of sea eagles detected relatively high levels of PFAS in eggs (Faxneld et al. 2014) and, of the 11 studies reporting PFAS concentrations in raptors that we reviewed (Table 2), a third used eggs. Addled and deserted eggs are also used as indicators of exposure to toxic metals, particularly Hg (Negro et al. 1993; Pain et al. 1999; Nygård 1999; Blanco et al. 2003). Laying females excrete approximately 20 % of their soft tissue methyl Hg (MeHg) into eggs (Lewis et al. 1993) and, because Hg occurs mainly as MeHg in eggs (Ackerman et al. 2013), total Hg can be used as a surrogate measurement for MeHg. Hg is not thought to cause significant eggshell thinning (Spann et al. 1972; Heinz 1974; Hill and Shaffner 1976) but MeHg is teratogenic in birds, increasing the incidence of malformations and embryonic mortality (Borg et al. 1969; Hoffman and Moore 1979; Heinz and Hoffman 2003; Frederick and Jayasena 2010). In contrast to Hg, little Pb is transferred from the female to eggs (Scheuhammer 1987; Furness 1993), although Pb may occur in eggshells as it acts as a calcium analogue (Pounds 1984; Lundholm and Mathson 1986; Scheuhammer 1987; Dauwe et al. 1999). Very little Cd is transferred to eggs of birds and often concentrations are below detection limits, regardless of the dietary levels of Cd consumed (Scheuhammer 1987; Furness 1996). Given this, it is perhaps surprising that a similar proportion of the egg contaminant studies we reviewed reported Pb and Cd as well as Hg (14–22 %; Table 2). This may perhaps simply reflect the potential for simultaneous determination of all three elements using inductively-coupled plasma mass spectrometry techniques, although this sometimes may be at a cost of reduced sensitivity for some elements.

### Feathers

Feathers are the sample type that are collected across more schemes and countries than any other sample type (Table 1) and so are potentially an important resource for contaminant monitoring. However, only 45 of the 249 raptor studies we reviewed (18 %) used feathers (Table 2). General attributes that make feathers useful for monitoring contaminants are: (i) they can be plucked in small numbers without causing permanent damage and so are minimally invasive; (ii) it is possible to pluck repeated samples from the same individual to assess changes over time; (iii) samples can also be collected from carcasses or as moulted feathers regardless of season, age or gender of the bird; (iv) samples are not readily degraded and can be transported and stored at room temperature; (v) samples are often available as historic samples from museum specimens, thereby providing opportunities to measure historic temporal trends in exposure. In addition, feathers can be used to measure biomarkers related to stress, such as fluctuating asymmetry and corticosterone levels (Bustnes et al. 2002; Bortolotti et al. 2008; Sillanpää et al. 2010; Strong et al. 2015).

All types of feathers can be used for contaminant analysis and as a tool for other studies, such as genetic studies (Speller et al. 2011; Presti et al. 2013). Choice of feather type can depend on the moulting pattern of the species, preening behaviour and the end point of the study. We reviewed 45 studies that analysed raptor feathers (Table 2) and 47 % used flight feathers, 44 % body feathers and 38 % tail feathers; more than one feather type was used in some studies. Both plucked and moulted feathers were analysed. When feathers have to be plucked (or cut close to the skin) from live birds, body feathers rather than flight feathers are normally used so as to avoid impairment of flight (Espín et al. 2014a); plucking or cutting feathers may also require a permit. Overall, we found that feathers plucked from live birds were used in about half as many studies as feathers plucked from dead birds found in the field or from museum collections (24 vs 56 % of the 45 studies we reviewed). Moulted feathers are easier to collect in the field than plucked feathers, but have the disadvantage that information on age, sex and body condition of the bird from which the feather has been shed, and the time of moult, is usually lacking; all can affect feather pollutant concentrations (García-Fernández et al. 2013a). Overall, use of moulted feathers (29 % of the 45 studies reviewed) appears to be similar to that of plucked feathers from live birds; in some studies moulted and plucked feathers have both been analysed. In some circumstances, analysis of moulted feathers may be the only way to monitor pollutant exposure, and feathers that fall into or just below the nest and look new are most probably from nesting parent birds.

Contaminants are deposited in feathers only while they are growing and there is no ongoing homeostasis between blood and feather concentrations, unlike between blood and internal tissues (Burger 1993; García-Fernández et al. 2013a). Despite this, several authors have found significant correlations between pollutant concentrations in feathers and in blood or internal tissues (Thompson et al. 1991; Dauwe et al. 2002; Jaspers et al. 2007a, 2011, 2013a; Eulaers et al. 2011a; Rajaei et al. 2011; Espín et al. 2014c). Moreover, some papers have described significant correlations between lead and cadmium concentrations in the feathers of adults and in the blood of their nestlings (Martínez-López et al. 2004, 2005). However, such associations are not always manifest (Dauwe et al. 2005; Jaspers et al. 2006, 2007b; Eagles-Smith et al. 2008; Espín et al. 2010a, 2012b), largely for two reasons. First, there may be significant temporal displacement between feather growth (and associated deposition of pollutant in the feather) and collection of internal tissues, during which time post feather-growth changes in diet and/or fat mobilization may alter internal tissue contaminant concentrations (García-Fernández et al. 2013a; Eulaers et al. 2014b). Second, there may be external contamination on the surface of the feather (Dauwe et al. 2003; Jaspers et al. 2008; Espín et al. 2010b; Cardiel et al. 2011), which in turn is affected by preening behaviour and by moult strategy. Moult is completed within one year in smaller species but can extend over two or more years in larger species (Hardey et al. 2006) and protracted moult can enhance inter-feather variability in age and the associated period over which external contamination can occur.

Analysis of recently grown feathers (such as those plucked from nestlings) or continuously replaced feathers reduces the issues both of temporal displacement and, to a large extent, external contamination (Jaspers et al. 2004). Where older feathers have to be used, variability due to external contamination can be reduced by restricting analysis to the shaft or rachis as external contamination tends to be greater for the vane than the shaft and the shaft is more easily and effectively cleaned (Jaspers et al. 2007a; Cardiel et al. 2011; Espín et al. 2012a; García-Fernández et al. 2013a). Overall though, many studies have primarily addressed the issue of external contamination of feathers by washing. This indeed may be the only viable option for museum feathers which may have been treated with arsenic, mercuric or insecticides compounds (Espín et al. 2014a; Sánchez-Virosta et al. 2015) or with organic preservatives that can change metals levels in feathers (Hogstad et al. 2003). In general, feathers are washed prior to analysis for metals with various combinations of deionised water, acetone, Triton X-100 and nitric acid (Burger and Gochfeld 2001; Dauwe et al. 2003; Jaspers et al. 2004; Cardiel et al. 2011; Espín et al. 2012a, 2014c) while distilled water tends to be used when analysing POPs (Jaspers et al. 2007a, b; Behrooz et al. 2009; Eulaers et al. 2011a, b, 2013, 2014a, b; Espín et al. 2012b). However, it has been argued that washing techniques tested to date are not effective in removing all external contamination (Jaspers et al. 2004, 2008, 2011) and further studies are needed to determine reliable methods for discriminating between internal and external contamination (Cardiel et al. 2011).

Concentrations of pollutants in feathers are typically expressed in ng/g feather. Bortolotti (2010) demonstrated that variation in mass and growth rate between feathers may affect the interpretation of contaminant concentrations. Therefore, feather concentrations should preferably be expressed as a function of deposition rate (García-Fernández et al. 2013a) in addition to a mass-based unit. The former is only possible when growth bars are visible in the feather (one pair of growth bars, dark and light, represents a 24-h period of growth), or when the growth rate of the feather is known (García-Fernández et al. 2013a); further studies are needed to determine the growth rate of feathers in some raptor species.

Feathers have been used to measure concentrations of all the major compounds that are monitored in raptors in Europe, except anticoagulant rodenticides (Table 2). They were originally mainly used for monitoring metals including Cd and Pb (Denneman and Douben 1993; Martínez-López et al. 2002, 2005; Dauwe et al. 2003), although there remains uncertainty over the suitability of feathers for monitoring environmental Cd concentrations. Some authors report that external contamination of feathers with Cd is minimal (Burger 1993; Ek et al. 2004) but others conclude that external contamination cannot be neglected (Pilastro et al. 1993; Dauwe et al. 2003; Jaspers et al. 2004). Cd concentrations in feathers have been correlated with those in internal tissues in some studies (Pilastro et al. 1993; Agusa et al. 2005) but not others (Nam et al. 2005; Orlowski et al. 2007). External contamination of feathers with Pb is known to be problematic. While feather Pb concentrations have been shown to be correlated with concentrations in internal tissues in juvenile birds (Golden et al. 2003), external Pb contamination after feather formation can elevate total feather Pb concentrations and is difficult to remove (Dauwe et al. 2002, 2003; Pain et al. 2005; Cardiel et al. 2011). Adult feathers subject to atmospheric Pb deposition for long periods may be strongly affected by external contamination (Franson and Pain 2011).

Mercury has also been extensively monitored in feathers (Lindberg and Odsjö 1983; Ortego et al. 2006; Espín et al. 2014c) and, like Pb and Cd, it is transferred from blood into feathers. MeHg binds with keratin uniformly along the feather (Thompson and Furness 1989a, b; Hahn et al. 1993; Dauwe et al. 2003; Misztal-Szkudlińska et al. 2012) and concentrations reflect those in blood during the period of feather growth (Lewis and Furness 1991). Unlike Pb, moult is a significant elimination pathway of Hg. Tissue Hg concentrations decline during moult and 70–93 % of the total Hg body burden can be sequestered in plumage (Burger et al. 1993). External contamination is typically small relative to total feather Hg burden, causing little spurious inter-feather variation (Lindberg and Odsjö 1983; Dauwe et al. 2003; Jaspers et al. 2004), and feather Hg concentrations tend to be correlated with residues in internal tissues (Thompson et al. 1991). Body contour feathers may be most representative of total body Hg burden as inter-feather variation is lower than in primaries (Furness et al. 1986). However, it has been suggested that feathers from nestlings are the best indicators of local Hg pollution (Solonen et al. 1999). Hg in the first down of chicks is derived from Hg in the egg while levels in developing non-down feathers reflect Hg concentrations in food given to the chick. Hg in both feather types therefore reflects Hg pollution in the diet of the provisioning adult birds. In addition, critical feather Hg concentrations that are associated with reproductive impairment (lower clutch and egg size, reduced hatching rate and decreased chick survival) have been suggested (NAS 1978).

More recently, there has been a focus on measuring legacy and emerging POPs in feathers (Jaspers et al. 2006, 2007b, 2011, 2013a; Meyer et al. 2009; Eulaers et al. 2011a, b; Herzke et al. 2011), although concentrations may be derived to a large extent from external contamination by preen oil (Jaspers et al. 2008). Washing techniques to remove preen oil that have been tested thus far may affect the internal load in the feather (Jaspers et al. 2008, 2011). However, external contamination with preen oil may be advantageous when using feathers for contaminant monitoring. This is because total feather concentrations of organic pollutants are increased by the lipid-rich preen oil and correlations between feather and internal tissue contaminant concentrations are enhanced (Jaspers et al. 2008; Solheim 2010; Eulaers et al. 2011b). Sampling feathers is also generally easier and less invasive than sampling preen oil. The degree of contamination of feathers with preen oil partly depends on preening frequency which varies with species, season, environmental conditions, and gender (van Iersel and Bol 1958; Greichus and Greichus 1974; Caldwell et al. 2001; Pap et al. 2010).

### Blood, plasma and serum

Raptors can be exposed to chemicals through inhalation, dermal contact and ingestion, although diet is considered to be the main exposure pathway for most major pollutants. Irrespective of exposure pathway, contaminants, once absorbed, are transported and distributed throughout the body via the blood. Contaminant half-lives are typically shorter in blood than in internal tissues (S.I. Table 2) and, depending on the timing of sampling relative to exposure, pollutant concentrations are generally lower and more variable than in body tissues. For lipid soluble contaminants such as POPs, low concentrations in blood are primarily related to relatively low lipid content compared to other commonly sampled tissues such as eggs and tissue (Elliott and Norstrom 1998). Overall, low contaminant concentrations in blood mean that relatively high volumes may be needed to achieve analytical detection limit and it may be necessary to obtain a greater degree of replication of samples to reduce the effects of relatively high inter-individual variability.

Overall, blood concentrations provide a non-destructive measure of recent exposure to compounds (Olsson et al. 2000; Martínez-López et al. 2009; Sonne et al. 2010; Eulaers et al. 2011b) but, because half-lives are relatively short, may not be indicative of medium or long-term exposure (Morrissey et al. 2010). Despite this, correlations between blood and internal tissue concentrations have been found for some compounds (García-Fernández et al. 1995, 1996, 1997; Henriksen et al. 1998; Eagles-Smith et al. 2008) but such relationships can be disrupted by remobilization of contaminants from fat or other depots during egg laying, moult, starvation and migration (Henny and Meeker 1981; Henriksen et al. 1996; Evers et al. 2005) and by variation in either exposure pattern or intrinsic factors such as age, size, and body condition (García-Fernández et al. 1995, 1997). Blood can also be analysed for effects biomarkers such as haematological parameters (including measurement of clotting performance), oxidative stress, plasma biochemistry, enzymatic activities, and gene expression (Nyholm 1998; Martínez-López et al. 2004; Fisher 2009; Cesh et al. 2010; Martinez-Haro et al. 2011a; Rattner et al. 2011, 2014b, 2015; Espín et al. 2014b, d; Maceda-Veiga et al. 2015).

Whole blood, plasma or serum can be used for contaminant analysis. Plasma contains fibrinogen, which results in coagulation unless the sample is treated with an anticoagulant (Ehresman et al. 2007), typically heparin or ethylenediamine tetraacetic acid (EDTA). Of the studies we reviewed, 42 measured pollutant concentrations in blood (Table 2) and heparin was used in 71 % and EDTA in 5 %; 24 % did not indicate which, if any, anticoagulant was used. EDTA can be problematic as it may affect clinical chemistry analytes and enzyme measurements (Hochleithner 1994) while significant movement of Pb from red cells to plasma can occur (deSilva 1981). However, heparin interferes with white blood cells and sometimes with PCR analysis, although heparinised plasma or serum is the recommended matrix for the vast majority of biochemical tests (Hochleithner 1994). Studies involving gene expression requires RNA conservation with RNAlater™ or similar preservatives (Maceda-Veiga et al. 2015).

Which blood compartment should be selected for analysis depends on the contaminant of interest and whether additional end-points (lipids, proteins, hormones) are to be measured. Advantages and disadvantages on using each compartment are summarised in Table 3. Use of whole blood may permit collection of smaller blood volumes which can be important when sampling small raptors (Volz et al. 2001). Most studies that have measured Pb in raptors have used whole blood (García-Fernández et al. 1995, 1997; Mateo et al. 1999; Benito et al. 1999; Pain et al. 2007; Gangoso et al. 2009; Espín et al. 2014b, d). Whole blood has also been recommended for analysing OCs and brominated flame retardants to avoid potential loss of contaminants in the cellular fraction (Volz et al. 2001; Leslie et al. 2013), although most lipid soluble contaminants are in the triglycerides present in the plasma fraction. Serum or plasma can be used for analysis of many contaminants but it is notable that concentrations of some poly- and perfluoroalkyl substances (PFASs) in whole blood are approximately half those in serum or plasma because of the volume displacement of cellular components, which do not appear to function as a sorbent for these substances (Ehresman et al. 2007). Of the 42 studies we reviewed that measured contaminant levels in raptor blood, whole blood was used in 79 % (to measure metals, POPs or anticoagulant rodenticides), plasma in 17 % (to analyse POPs, PFAS and pharmaceuticals), and serum in 5 % (analysis of POPs).

**Table 3:** Advantages and disadvantages on the use of the different blood compartments for pollutant exposure monitoring of raptors

Blood compartment	Advantages	Disadvantages
Whole blood	Small blood volumes can be collected Allows analysis for the broadest spectrum of contaminants Higher concentrations of some contaminants (decaBDE) are present because of their partition to whole blood compartments Can be used for measurement of haematology, blood cell counts and some biomarkers Can be used for gene expression studies	Anticoagulants are needed Lower concentrations of some contaminants (PFASs) because of cellular components RNA preservatives are needed
Plasma/Serum	Measurement of biochemistry, antibodies and enzymatic activities Higher concentrations of some contaminants (PFASs)	Anticoagulants are needed (plasma) Samples have to be centrifuged Plasma/serum separation may produce loss of some contaminants (decaBDE, OCs)
Red blood cells	Measurement of antioxidant molecules and oxidative damage on lipids, proteins and DNA	Samples have to be centrifuged Erythrocytes may need to be washed using saline solution for some analyses Concentration of some compounds may be lower
Dried blood spot	Anticoagulants are not needed Simplify sampling Facilitate handling, storage and transport of blood samples	Limited number of laboratories mastering sufficiently sensitive methods Variable amount of trace metals in the filter paper (both within and between lots) and it is possible external contamination

Dried blood spots (DBSs) have been proposed as an alternative to fresh blood for monitoring chemical residues in raptors (Shlosberg et al. 2011a, b). The possibility of quantifying POPs, metals and PFASs in small (~ 200 µl) blood volumes could simplify sampling as this could be done by venepuncture with microhematocrit capillaries rather than larger syringes and so be less invasive. Moreover, DBS are likely to be easier to handle, store and transport than blood samples. However, to date, the number of laboratories with sufficiently sensitive methods to analyse DBS is limited. In addition, Stove et al. (2012) have identified several limitations to the technique, such as the presence of variable amounts of trace metals in the filter paper (within and between production batches) and possible external contamination.

In terms of expression of contaminant data, blood concentrations are typically expressed on a wet weight basis or by volume. Conversion factors (wet weight to volume) of 0.94 (whole blood) and 0.98 (plasma) have been suggested, based on relative density (Coeurdassier et al. 2012), assuming whole blood and plasma densities of 1.060 and 1.025 g ml^−1^, respectively (Barlow and Whitehead 1928). Blood lipid levels in birds may change seasonally (deGraw et al. 1979) and with body condition (Elliott et al., 1998). Reporting of concentrations on both wet and lipid weight basis is recommended for lipid soluble POPs.

Overall, blood has been used to monitor concentrations of all the main priority contaminants in raptors but, in the studies we reviewed, analysis was mainly for POPs and toxic metals, particularly Pb (Table 2). Blood metal concentrations have been widely related to effects that range from sub-lethal (such as enzyme inhibition) through to mortality (Czirjak et al. 2010; Franson and Pain 2011; Shore et al. 2011; Wayland and Scheuhammer 2011; Espín et al. 2015) and blood may be particularly useful for monitoring Hg in raptors (Henny and Elliott 2007; Guigueno et al. 2012). This is because, as with eggs, Hg in blood is almost entirely present as MeHg (DesGranges et al. 1998; Fournier et al. 2002; Rimmer et al. 2005) and total Hg can be used as a [cheaper] surrogate measure for MeHg (Eagles-Smith et al. 2008); Hg concentrations in blood are highly correlated with MeHg in liver (Henny et al. 2002).

### Internal tissues

Pollutant concentrations in internal tissues are a key indicator of bioaccumulation. Tissue samples are collected across some 20 countries in Europe (Table 1, S.I. Fig. 1), but is only possible where carcasses are found in the field or injured birds are euthanasied for welfare reasons. Which specific internal tissue should be analysed for contaminants depends in part on the endpoint of the study, the toxicokinetics of the compounds of interest (García-Fernández et al. 2008) and the target organ for toxicity. Pollutant concentrations associated with adverse effects in target organs, or with specific endpoints such as mortality and reproduction, have been extensively reported (Beyer and Meador 2011). However, tissues which accumulate the highest contaminant concentrations and/or multiple contaminants that can be determined simultaneously are sometimes analysed in preference to the target organ. In our review of 249 European studies, 111 analysed internal tissues and the liver was by far the most commonly analysed organ (98 studies, Table 2). In some cases, internal tissues are collected specifically for the determination of effect biomarkers (Mateo et al. 2003; Rainio et al. 2012) but these are labile, must be measured or conserved immediately after biopsy or death (Mateo et al. 2003; Rainio et al. 2012) and cannot be measured in tissues from carcasses found in the field some time after death.

Post-mortem decomposition and cause of death can both potentially affect tissue contaminant concentrations. Decomposition may lead to desiccation of tissues and microbial post-mortem metabolism of contaminants (Ferner 2008; Butzbach 2010). Although time of death can be estimated (Payne-James et al. 2003), we are unaware of verified methods that can be used to normalise contaminant concentrations for extent of decomposition. Cause of death can alter tissue contaminant concentrations in that starvation can deplete adipose tissue. The associated metabolism of fat leads to remobilisation of lipophilic compounds which are subsequently distributed via the bloodstream to highly metabolically active organs such as the liver (Hela et al. 2006). This can result in elevated liver contaminant concentrations, an effect exacerbated by starvation-induced liver wastage (Wienburg and Shore 2004; Crosse et al. 2013). Body condition can be quantified during necropsy (Espín et al. 2014a) and either included as a variable in any statistical comparisons of contaminant data, or used as a factor to focus sample selection so that source of variance is minimised (for example, Elliott et al. 2015).

Pollutant concentrations in internal organs are expressed on a wet, dry or lipid weight basis and this variability can hamper inter-study comparisons (Henny and Elliott 2007). In general, metal concentrations are reported on dry weight basis to avoid spurious variation caused by differences in water content between samples. Concentrations of lipophilic pollutants are often reported on a wet weight or lipid weight basis to normalise for inter-individual and inter-species variation in organ lipid content. Wet concentrations can be converted to a dry weight and lipid weight basis (S.I. Document 4) but the water and lipid content of tissues must be known.

In terms of analysis of particular compound groups, organic contaminants typically bioaccumulate strongly in adipose fat depots and in those body tissues with relatively high lipid content, such as liver, kidney, muscle and brain (Borlakoglu et al. 1991; Buckman et al. 2004; Voorspoels et al. 2006; Espín et al. 2010a). Partitioning between tissue types varies with physico-chemical properties and compounds with log K_ow_ < 3.3 have a higher affinity for phosphatidylcholine while those with log K_ow_ > 3.3 partition preferentially accumulate into trioleoylglycerol (Sandermann 2003). This may account for why more polar OCs (for example lindane-γ-HCH) preferentially accumulate in tissues like the brain which contains high concentrations of more polar lipids such as phospholipids. Non-polar compounds (for example DDTs and PCBs) are mainly accumulated in organs such as liver or adipose tissue that contain high proportion of triglycerides (Kawai et al. 1988; Noble and Cocchi 1990; Sandermann 2003; Maervoet et al. 2005). Overall though, the highest absolute concentrations of many non-polar organic compounds are found in fat (María-Mojica et al. 2000; Kenntner et al. 2003b; Espín et al. 2010a), probably reflecting the affinity of these compounds for triglycerides. Therefore, when carcasses are available, adipose tissue can be useful for monitoring chronic exposure (Kenntner et al. 2003b; Hela et al. 2006), although only 9 % of 137 studies on POPs that we reviewed reported concentrations in fat (Table 2). This may be partly because sampling fat can be difficult when carcasses are partially decomposed or when body condition is poor.

Liver POP concentrations often reflect recent dietary exposure (Newton et al. 1993; Kenntner et al. 2003b; van Drooge et al. 2008), although concentrations of lipophilic compounds can be elevated by redistribution of compounds from depleting fat stores (Kenntner et al. 2003a; Wienburg and Shore 2004; Crosse et al. 2013). Less lipophilic compounds such as PFASs also accumulate in liver [and other soft tissues] where they bind to protein albumin (Jones et al. 2003; Yoo et al. 2009; Stahl et al. 2011). Studies in leghorn chickens (*Gallus gallus*) suggest that the highest concentrations of perfluorooctane sulfonate (PFOS) and perfluorooctanoic acid (PFOA), two PFASs widely used in industry (ATSDR 2009), occur in the liver and kidney, respectively (Yoo et al. 2009). Liver concentrations for a range of organic contaminants can be related to those estimated to be indicative of adverse effects (Blus 2011; Elliott and Bishop 2011; Harris and Elliott 2011), while effects on cytochrome P450 activity and antioxidant enzymes in individuals can be measured directly if samples are fresh (Helgason et al. 2010). However, when the primary interest concerns the assessment of POP-induced effects, analysis of the brain can be informative as it is a major target organ and concentrations associated with mortality and chronic effects have been defined for various compounds (Blus 2011; Elliott and Bishop 2011; Harris and Elliott 2011). However, the brain was analysed in only about 5 % of the POPs studies that we reviewed (Table 2). This is maybe because, in more recent years, lethal exposure to POPs is rarely encountered and, furthermore, excision of an intact brain from a bird skull is time consuming compared with removal of other tissues such as the liver.

The liver and kidneys are the internal organs most typically used for analysis of metals. Approximately 30–45 % of studies in which Pb, Cd and/or Hg were determined involved analysis of these organs (Table 2); Pb is also often analysed in bone (24 % of studies). The relative distribution of these metals between different body tissues differs markedly.

Pb follows a tri-compartmental kinetic model where bone is the principle organ of accumulation (García-Fernández et al. 1997), but there is also distribution to other major organs, primarily liver, kidney and also brain (Custer et al. 1984; Franson and Pain 2011). Pb concentrations tend to be higher in the kidneys than in the liver and highest in bone when exposure is chronic or less recent (García-Fernández et al. 1995) and often highest in the liver at acute poisoning (e.g. Helander et al. 2009). Overall, bone Pb reflects lifetime accumulation while soft tissue and blood residues are indicative of, and can be used to monitor, shorter-term exposure (García-Fernández et al. 1995, 1997). Threshold concentrations in these organs for adverse effects have been suggested for Falconiformes (Franson and Pain 2011).

The distribution of Cd between body tissues is dose-dependent (Scheuhammer 1987). The kidney is the main organ for accumulation during chronic exposure to low concentrations, but accumulation is greater in liver following acute exposure to high doses (García-Fernández et al. 1995, 1996). The liver:kidney Cd concentration ratio can thus be used as an indicator of exposure pattern (Scheuhammer 1987). Nevertheless, in contaminated areas, liver Cd may be the better indicator of chronic exposure since kidney concentrations fall significantly with onset of Cd-induced tubular dysfunction (Scheuhammer 1987) and so may not be representative of the true levels of exposure. Normally though, Cd concentrations in birds are at least two orders of magnitude below concentrations associated with clinically-manifest kidney damage (Wayland and Scheuhammer 2011) and, in such habitats, the kidney may be a more useful monitor of exposure as concentrations are likely to be higher and more likely to exceed analytical limits of detection.

For Hg, the highest tissue concentrations are found in the kidney and liver, followed by muscle and brain (Norheim and Frøslie 1978; Holt et al. 1979; Häkkinen and Häsänen 1980; Kalisinska et al. 2014). How Hg partitions between internal organs depends on whether exposure is to organic or inorganic forms of the metal, and the Hg kidney:liver molar ratio has been proposed as a mean of distinguishing between chronic exposure to MeHg and inorganic Hg (Scheuhammer 1987). Kidney:liver molar ratios much higher than 1 reflect exposure to inorganic Hg, whereas a ratio closer to 1 (and < 2) is characteristic for exposure to MeHg. In general, the liver and/or the kidney can be used for monitoring trends in exposure. Threshold liver and kidney concentrations associated with adverse effects in birds generally have been suggested (Shore et al. 2011) but interpretation of internal tissue concentrations is problematic. This is because of demethylation processes and sequestration of selenium (Se) bound inorganic Hg (Henny et al. 2002; Eagles-Smith et al. 2008), Se forming a complex with Hg and protecting against Hg toxicity (Scheuhammer 1987; Cuvin-Aralar and Furness 1991; Scheuhammer et al. 2008). Ideally, total Hg, MeHg and Se concentrations should be measured simultaneously and analysis of both liver and kidney can provide information on the nature of exposure.

Of the remaining priority compounds outlined in the current paper, the liver is the tissue which is almost always used for analysis of anticoagulant rodenticides (Table 2; Rattner et al. 2014a). These, and in particular the second-generation anticoagulant rodenticides, are sequestered by high affinity binding sites in the liver (Huckle et al. 1989; Vandenbroucke et al. 2008; Fisher 2009). Liver half-lives vary between compounds (Vandenbroucke et al. 2008; S.I. Table 2) but, overall, liver concentrations provide a signal of single or multiple exposures integrated over periods of weeks and months (Shore et al. 2015). Rodenticide concentrations tend to be higher in liver than in other tissues (Huckle et al. 1988, 1989) and extent of rodenticide use, diet, resistance in rodents and physiology can all affect the magnitude of residues accumulated by raptors (Shore et al. 2015). There is no clear diagnostic or threshold liver concentration that is associated with mortality but higher residues are associated with a greater probability of mortality (Thomas et al. 2011).

### Other sample matrices

Although less frequently used, other matrices such as crop contents, regurgitated pellets, excrement, preen oil and talons can be analysed for contaminants. Recommendations on sampling methods are given by Espín et al. (2014a).

Crop contents can be obtained from carcasses and regurgitated pellets from roost and nest sites. Both provide information about spatio-temporal variation in diet which can be important when assessing likely exposure of raptors to contaminants (Mañosa et al. 2003; van Drooge et al. 2008). Crop (and gizzard/stomach) contents have been used to determine exposure to anticoagulant rodenticides (Ruder et al. 2011) and to confirm the occurrence of lethal poisoning by organophosphate and carbamate insecticides (Elliott et al., 1996; 1997) and compounds such as strychnine, metaldehyde and paraquat (Berny 2007). Regurgitated pellets are frequently used in dietary studies (Love et al. 2000; Redpath et al. 2001; Terraube et al. 2011; Trierweiler and Hegemann 2011) and are widely collected across Europe by numerous raptor population monitoring schemes (Table 1, S.I. Fig. 1). Pellets also have characteristics that make them well-suited for use as non-invasive indicators of environmental contaminants. These include that a large number of samples can be obtained, samples can be collected from species that are difficult to trap or that are rarely found as carcasses, and pellets can be collected regardless of the age or gender of the bird or of season. Pellets have been used to measure exposure of raptors to toxic metals (Bostan et al. 2007; Lopes et al. 2010) including Pb from shot, the presence of which can be confirmed by X-rays (Mateo et al. 2001; Pain et al. 2007). Chemical analysis has also been used to assess exposure of owls to second-generation anticoagulant rodenticides (Gray et al. 1994; Newton et al. 1994; Eadsforth et al. 1996) and regurgitated barn owls (*Tyto alba*) pellets were estimated to contain 25–29 % of ingested dose (Gray et al. 1992, 1994; Newton et al. 1994).

Sampling of excrement can be either minimally invasive or non-invasive as fresh samples can be obtained by inducing birds to defecate when they are being handled or by collecting faeces from around nests and roosts. Large amounts of sample can normally be obtained quickly, although DNA analysis may be needed to identify the species and/or individual that produced the sample (Cheung et al. 2009). Excrement has been used in studies on diet composition, hormones and genetics (Goymann and Jenni-Eiermann 2005; Waits and Paetkau 2005; Carlisle and Holberton 2006; Reynolds et al. 2006) and when measuring effects biomarkers such as porphyrins (Akins et al. 1993; Mateo et al. 2006; Martinez-Haro et al. 2011b). While there have been a number of avian contaminant studies that have analysed faecal material, they have generally not been on raptors (Howald 1997; Beyer et al. 1998; Dauwe et al. 2000; Sun and Xie 2001; Sun et al. 2005, 2006; Yin et al. 2008; Costa et al. 2012; Berglund et al. 2015). Contaminant concentrations in excrement have been considered particularly useful as an indicator of exposure to contaminants that are poorly absorbed across the gut. Such compounds include non-essential metals (Dauwe et al. 2000, 2004; Morrissey et al. 2005), which are typically present in higher (and more easily detected) concentrations in excrement than in food items (Morrissey et al. 2005; Yin et al. 2008). However, there appears to be a lack of any direct relationship between concentrations in faeces and in internal tissues for the majority of metals, suggesting that faecal measurements may not be a suitable proxy for accumulation (Berglund et al. 2011).

In contrast to faeces, preen oil provides information about pollutants that have been absorbed into the body. It is an oily secretion of waxes from the uropygial gland, a sebaceous gland at the base of the tail feathers. Birds use this oil when preening to protect and waterproof their feathers (Solheim 2010). Collection of preen oil can be considered non-invasive when sampled from carcasses and minimally invasive when collected from living birds as it is necessary to press the gland softly to expel the oil (Espín et al. 2014a). To date, preen oil is only rarely collected by raptor monitoring schemes in Europe (Table 1, S.I. Fig. 1) and can sometimes be difficult to sample in sufficient quantity for analysis, particularly from nestlings (Eulaers et al. 2011b). Uropygial secretion quantity and composition also varies between species (Campagna et al. 2011; García-Fernández et al. 2013a). The oil can contain high concentrations of lipophilic pollutants (van Den Brink 1997; Yamashita et al. 2007; Jaspers et al. 2008, 2011; Eulaers et al. 2011b), which is related to its high lipid content (87 % lipid content, Eulaers et al. 2011b), and is considered an excretion route for lipophilic compounds (Solheim 2010). Contaminant concentrations in preen oil are correlated with those in internal tissues (van Den Brink 1997; Eulaers et al. 2011b), but compound profiles can differ. For example, preen oil is relatively richer in lower-chlorinated PCB congeners (di-, tri-, and tetra-CB) than internal tissues (Larsson and Lindegren 1987; Yamashita et al. 2007; Jaspers et al. 2013b).

Clipped talons are keratinized tissues that, during their formation, can accumulate certain contaminants. To our knowledge, only Hopkins et al. (2007) have used talons to analyse contaminant concentrations in bird species. They found Hg concentrations in clipped talons from ospreys (*Pandion haliaetus*) were correlated with concentrations in internal tissues and the association was stronger than that between feathers and soft tissues. This was believed to be because talons, unlike feathers, grow continuously, and talon Hg concentrations are more likely to be representative than feathers of the current Hg body pool. Hopkins et al. (2007) collected a limited portion of talon (5 mm length from digits 2 and 3 on the left foot) without disturbing the talon’s blood supply but it is arguable as to whether this could be classed as a non-invasive sample as talons are important for catching, holding and processing prey, and clipping the talons of live wild raptors may adversely affect hunting performance. Collection from carcasses is possible but may only be advantageous when internal organs are too decomposed for analysis.

## Monitoring other contaminants of current and emerging concern

This review has focussed on the suitability of different matrices for the compounds that are most commonly monitored across Europe. However, there are also other current and emerging compound groups of concern either in terms of general environmental contamination or as a direct threat to raptors themselves.

Pharmaceuticals and personal care products, including human and veterinary drugs, fragrances, and cosmetics, are produced in high volumes and are widely used (Rüdel et al. 2006; Nakata et al. 2007). Some compounds, such as synthetic musks, are highly lipophilic and have been detected in several aquatic organisms (Nakata et al. 2007), and pharmaceuticals generally have been detected in the environment across the world (Boxall et al. 2012). There is also growing concern over the potential ecotoxicological effects of releasing veterinary pharmaceuticals from animal feed lot operations into streams and estuaries (Daughton and Ternes 1999; Kolpin et al. 2002). The potential for monitoring environmental concentrations of pharmaceuticals using raptors has recently been investigated in ospreys (*Pandion haliaetus*) (Lazarus et al. 2015), and has been more widely considered by Shore et al. (2014), but generally data remain scarce. Two exceptions however are non-steroidal anti-inflammatory drugs (NSAIDs) such as e.g. diclofenac, and veterinary drugs used as antiparasitics (organophosphate and carbamate compounds) or to euthanase animals (e.g., barbiturates).

NSAIDs have been studied most intensively in raptors because of their catastrophic impacts on vulture populations (Green et al. 2004, 2006, 2007; Oaks et al. 2004; Naidoo et al. 2009; Sharma et al. 2014; Galligan et al. 2014; Cuthbert et al. 2014; Zorrilla et al. 2015). This work has focussed largely on Asian and African populations although, in Spain, a wild Griffon vulture (*Gyps fulvus*) has been found to be exposed to and apparently killed by the NSAID flunixin (Zorrilla et al. 2015), and other pharmaceuticals have been detected in other individuals (García-Fernández et al. 2013b). NSAIDs bind strongly to plasma protein and are metabolised mainly in the liver, usually to inactive compounds (Lees et al. 2004). Plasma, liver and kidney can all be used to monitor NSAID exposure and can also be used for effects assessment, since diclofenac-treated vultures have shown an increase in plasmatic uric acid, alanine transferase and glutamic pyruvate transaminase, and histopathological changes in kidneys and liver (Swan et al. 2006; Jain et al. 2009); visceral gout has also been a key indicator of exposure (Cuthbert et al. 2015). Data regarding the safety of other NSAIDs and risks to other species is currently limited (Taggart et al. 2009; García-Fernández 2014; Sharma et al. 2014); and further monitoring of NSAIDs concentrations in raptor populations and evaluation of potential effects therefore remains a priority.

Organophosphate (OP) and carbamate (CB) compounds are used as antiparasitics in livestock and secondary exposure can result in raptor mortalities (Henny et al. 1985; Mineau et al. 1999). These compounds are rapidly metabolized in the body and so the proventriculus, gizzard or gastrointestinal content are the samples commonly used to determine exposure, although residues have been also detected in liver, brain and muscle (Hamilton and Stanley 1975; Glaser 1999; Berny 2007; Shimshoni et al. 2012; Mateo et al. 2015). The toxicity of OP and CB pesticides is mainly due to the disruption of the nervous system by inhibition of cholinesterase (ChE) enzymes activity (Glaser 1999; Hill 2003), and measurement of brain ChE in dead birds and plasma/serum ChE activity in live birds is also used as biomarker to monitor exposure (Elliott et al., 1996; 1997; Martínez-Haro et al. 2007; Shimshoni et al. 2012). Among other veterinary drugs of concern, barbiturates, of which pentobarbital is the predominant active component, are commonly used to euthanize domestic animals (Aldeguer et al. 2009). Secondary barbiturate poisoning has been reported in raptors (Shore et al. 2014) and pentobarbital and, some other euthanasia drugs, can be detected by analysis of the liver or upper gastrointestinal contents. Detection of residues in the liver provides more definitive evidence that a drug has been absorbed from the ingesta, as do blood samples from live birds (Thomas 1999).

A newer class of insecticides now in prevalent use worldwide and which has attracted attention because of risk to pollinators (Rundlöf et al. 2015), aquatic organisms (Morrissey et al. 2015) and insectivorous birds (Mineau and Palmer 2013; Hallmann et al. 2014) are the neonicotinoid pesticides. These insecticides act as agonists of the insect nicotinic acetylcholine receptors (AChRs) (Gibbons et al. 2014) and raptor prey species that feed on seed and crops may be exposed to these pesticides. Experimental studies have shown that neonicotinoid pesticides may produce adverse effects on reproduction, behaviour, immune system, growth and development in birds (Cox 2001; Mineau and Palmer 2013; Tokumoto et al. 2013; Lopez-Antia et al. 2013, 2015) but little is known about the exposure in top predators, including raptors. The liver and crop content can be analysed to determine exposure to neonicotinoid pesticides in dead birds (Lopez-Antia et al. 2015), and it may be possible to use blood as a non-destructive sample to determine exposure, as has recently been demonstrated in humans (Mohamed et al. 2009; Yeter and Aydın 2014; Yamamuro et al. 2014). Experimental studies on rats have shown that the neonicotinoid imidacloprid inhibits δ-ALAD activity in liver tissue (Sauer et al. 2014), causes changes in antioxidant enzymes in brain and liver, and increases lipid peroxidation in plasma, brain and liver (El-Gendy et al. 2010; Duzguner and Erdogan 2012) while red-legged partridges (*Alectoris rufa*) exposed to imidacloprid suffered sublethal effects including altered biochemical parameters in plasma and oxidative stress in red blood cells (Lopez-Antia et al. 2013). Therefore, blood, liver and brain can be used for monitoring both exposure and effects from these compounds. None of the programmes surveyed by Gómez-Ramírez et al. (2014) specified that they monitored exposure to neonicotinoids in raptors but such monitoring would help determine if there may be any significant food-chain transfer and exposure in raptors.

## Conclusions

In this paper, we have reviewed the merits of different sample matrices for measuring environmental contaminants in raptors. There is no optimal all-purpose tissue. The scientific relevance of each sample type depends on the aims and objectives of any study and the compound(s) of interest, but we have attempted to show the relative merits of each matrix for major groups of contaminants. Of all the matrices, blood and liver are probably used most extensively for determining recent and longer-term exposure, respectively. However, blood samples are less widely and frequently collected across Europe than several other matrices (Table 1) and volume size and storage requirements are likely to limit the number of analyses that can be conducted. Internal tissue and carcass collection is conducted by more schemes than for blood but in fewer countries (Table 1), and we have no information on the relative numbers of each tissue type collected or their typical condition. Where blood and/or liver can be collected, they are likely to be the most useful for monitoring but developing techniques for a wider range of matrices is merited so that use of limited blood and liver samples can be focussed towards compounds where use of other matrices is not appropriate. This may require greater use of material that to date is widely collected but relatively little analysed, such as food remains and regurgitated pellets (Tables 1 and 2), and enhanced routine collection of other samples, such as preen oil. The potential of such samples may increase in the future as analytical sensitivity and understanding of contaminant toxicokinetics further improves.

Although a wide range of samples are collected across Europe, (failed) eggs and feathers are the two matrices for which collection effort appears to be greatest (Table 1). These may provide the best opportunities for instigating widescale (pan-continental) monitoring, although neither is suitable for all contaminants. Eggs are already widely used and critical concentrations have been suggested for various compounds (Beyer and Meador 2011) but data need to be reported in a harmonised way (for example, correcting for desiccation). Recently grown feathers can be used for determining metals, and potentially for organic contaminants because of the presence of preen oil on the feathers, but methodologies to distinguish external from internal contamination need to be employed and care taken in interpreting resultant data. A further major challenge for large-scale monitoring using any matrix is that samples are unlikely to be available for the same species over a range of habitats across large spatial areas. Approaches using species guilds, where guilds are defined by similarity in trophic strategy, may be needed to obtain such coverage. Developing this concept, and the data needed to characterise the within-guild read-across between species for specific sample matrices, together with better harmonisation between analytical laboratories to ensure data comparability, are perhaps the next steps needed to develop widescale contaminant monitoring using raptors.

## Electronic supplementary material

Below is the link to the electronic supplementary material.

Supplementary material 1 (DOCX 1062 kb)
