# Supplementary material for: Tracking pan-continental trends in environmental contamination using sentinel raptors—what types of samples should we use?
Source: Ecotoxicology. 2016 Mar 5;25:777–801. doi: 10.1007/s10646-016-1636-8 (PMC4823350; doi:10.1007/s10646-016-1636-8)
Supplement: Supplementary file 1 — Supplementary material 1 (DOCX 1062 kb) [file 10646_2016_1636_MOESM1_ESM.docx]

**Electronic Supplementary Information**

**Tracking pan-continental trends in environmental contamination using sentinel raptors—what types of samples should we use?**

Espín S^1,2^*, García-Fernández AJ^1^, Herzke D^3^, Shore RF^4^, van Hattum B^5,16^, Martínez-López E^1^, Coeurdassier M^6^, Eulaers I^7,14^, Fritsch C^6^, Gómez-Ramírez P^1^, Jaspers VLB^7,8^, Krone O^9^, Duke G^10^, Helander B^11^, Mateo R^12^, Movalli P^13^, Sonne C^14^, van den Brink NW^15^.

^1^Department of Toxicology, Faculty of Veterinary Medicine, University of Murcia, Campus de Espinardo, 30100 Murcia, Spain; ^2^Section of Ecology, Department of Biology, University of Turku, 20014 Turku, Finland; ^3^Norwegian Institute for Air Research, FRAM—High North Research Centre for Climate and the Environment, 9296 Tromsø, Norway; ^4^NERC Centre for Ecology and Hydrology, Lancaster Environment Centre, Library Avenue, Bailrigg, Lancaster LA1 4AP, UK; ^5^Institute for Environmental Studies, VU University, De Boelelaan 1087, 1081 HV Amsterdam, The Netherlands; ^6^Chrono-Environnement, UMR 6249 University Bourgogne Franche-Comté/CNRS Usc INRA, 16 Route de Gray, 25030 Besançon Cedex, France; ^7^Behavioural Ecology and Ecophysiology group, Department of Biology, University of Antwerp, Universiteitsplein 1, Wilrijk 2610, Belgium; ^8^Department of Biology, Norwegian University of Science and Technology, EU2-169, Høgskoleringen 5, Trondheim, 7491, Norway; ^9^Leibniz Institute for Zoo and Wildlife Research, Alfred-Kowalke-Strasse 17, 10315 Berlin, Germany; ^10^Oxford University Environmental Change Institute, Centre for the Environment, South Parks Road, Oxford, OX1 3QY, UK; ^11^Environmental Research & Monitoring, Swedish Museum of Natural History, Box 50007 SE-104 05 Stockholm, Sweden; ^12^Instituto de Investigación en Recursos Cinegéticos-IREC (CSIC-UCLM-JCCM), Ronda de Toledo s/n, 13071 Ciudad Real, Spain; ^13^Naturalis Biodiversity Center, Department of Collections, Darwinweg 2, 2333 CR Leiden, The Netherlands; ^14^Århus University, Department of Bioscience, Artic Research Centre (ARC), Frederiksborgvej 399, PO Box 358, DK-4000 Roskilde, Denmark; ^15^Division of Toxicology, Wageningen University, PO Box 8000, NL-6700EA, Wageningen, The Netherlands; ^16^Deltares, Marine and Coastal Systems, P.O. Box 177, 2600 MH Delft, The Netherlands.

*Corresponding author: Silvia Espín. Telephone: +34 868884317/ +358 23336006. E-mail address: [silvia.espin@um.es](mailto:silvia.espin@um.es), [sieslu@utu.fi](mailto:sieslu@utu.fi)

This information is in support of an article published in *Ecotoxicology*.

**S.I. Document 1. Details of the methods by which literature was searched.**

The search of the literature available was conducted using different databases including PubMed, Science Direct, Springer, and Web of Science. Different keywords and combinations of terms were used, such as “birds of prey”, “raptors”, “predatory bird”, “contaminants”, “pollutants”, “heavy metals”, “trace elements”, “Pb”, “Cd”, “Hg”, “organohalogenated compounds”, “organochlorines”, “pesticides”, “organic pollutants”, “polychlorinated biphenyls”, “PCBs”, “flame retardants”, “polybrominated diphenyl ethers”, “PBDEs”, “polyfluoroalkyl compounds”, “perfluoroalkyl compounds”, “perfluorinated contaminants”, “PFASs”, “PFCs”, “anticoagulant rodenticides”, “neonicotinoid”, “pharmaceuticals”, “NSAIDs”, “diclofenac”. Several Google searches were also done to look for reports of projects and other documents that are not available in the major databases. In addition, all coauthors of this paper checked the list of references in the database to identify additional documents on the issue that had been missed. A database with detailed information about the literature we reviewed is available online ([www.eurapmon.net](http://www.eurapmon.net)) and a summary of this database is presented in Table 2 and Supplementary Information (S.I.) Table 3.

**S.I. Document 2. Shell thickness calculation.**

An index of eggshell thickness is usually calculated from shell mass and egg dimensions (Schönwetter 1960; Ratcliffe 1967; Moriarty et al. 1986; Nybø et al. 1997), and a single direct measurement is often made through the hole used to blow eggs or an average is calculated from repeat measurements along the long axis of cut eggs. Blowhole and cut-egg measurements of shell thickness are highly correlated, as are direct measures and indices of eggshell thickness, and shell thickness at the equator of the egg is thought to serve as a good index of average shell thickness for a broad range of species (Maurer et al. 2012).

References

Maurer G, Portugal SJ, Cassey P (2012) A comparison of indices and measured values of eggshell thickness of different shell regions using museum eggs of 230 European bird species. Ibis 154:714–724. doi: 10.1111/j.1474-919X.2012.01244.x

Moriarty F, Bell AA, Hanson H (1986) Does p,p′-DDE thin eggshells? Environmental Pollution Series A, Ecological and Biological 40:257–286. doi: 10.1016/0143-1471(86)90100-5

Nybø S, Staurnes M, Jerstad K (1997) Thinner Eggshells of Dipper (*Cinclus Cinclus*) Eggs from an Acidified Area Compared to a Non-Acidified Area in Norway. Water, Air, & Soil Pollution 93:255–266. doi: 10.1023/A:1022110321515

Ratcliffe DA (1967) Decrease in Eggshell Weight in Certain Birds of Prey. Nature 215:208–210. doi: 10.1038/215208a0

Schönwetter M (1960) Handbuch der Oologie., Meise, W. (eds). Akademie Verlag, Berlin.

**S.I. Document 3. Calculation of fresh weight and desiccation index of eggs.**

The weight of the egg when freshly laid is calculated from the linear dimensions of the egg (Hoyt 1979), ideally using a species-specific weight coefficient which can be calculated from Schönwetter (1960).

W = K_W_*LB^2^; W = fresh weight, K_W_ = weight coefficient, L = length, B = breadth

A desiccation index (Di) can also be calculated:

Di = (W - S) / V; W = weight (g) of the sampled egg, S = dry shell weight (g) and V = inside volume (ml) of the egg; Helander et al. 2002). The outside egg volume can be calculated from the linear measurements of the egg, adjusted for eggshell thickness to obtain the inside volume (for further details see Helander et al. 1982, 2002). The outside egg volume can also be measured directly by water displacement.

References

Helander B, Olsson A, Bignert A, et al (2002) The role of DDE, PCB, coplanar PCB and eggshell parameters for reproduction in the white-tailed sea eagle (*Haliaeetus albicilla*) in Sweden. Ambio 31:386–403.

Helander B, Olsson M, Reutergårdh L (1982) Residue Levels of Organochlorine and Mercury Compounds in Unhatched Eggs and the Relationships to Breeding Success in White-Tailed Sea Eagles *Haliaeetus albicilla* in Sweden. Holarctic Ecology 5:349–366. doi: 10.2307/3682220

Hoyt DF (1979) Practical methods of estimating volume and fresh weight of bird eggs. Auk 96:73–77.

Schönwetter M (1960) Handbuch der Oologie., Meise, W. (eds). Akademie Verlag, Berlin.

**S.I. Document 4. Conversion of contaminant concentrations in wet weight to a dry weight and lipid weight basis.**

Concentration dw = Concentration ww * 100 / (100 - % moisture)

Concentration lw = Concentration ww * 100 / % lipid content

**S.I. Figure 1**. Maps of European countries showing the number of schemes that collect each type of sample from raptor species. This figure is based on data from 281 monitoring schemes from 35 countries that answered questionnaires about their existing monitoring activities.

Blood Feathers


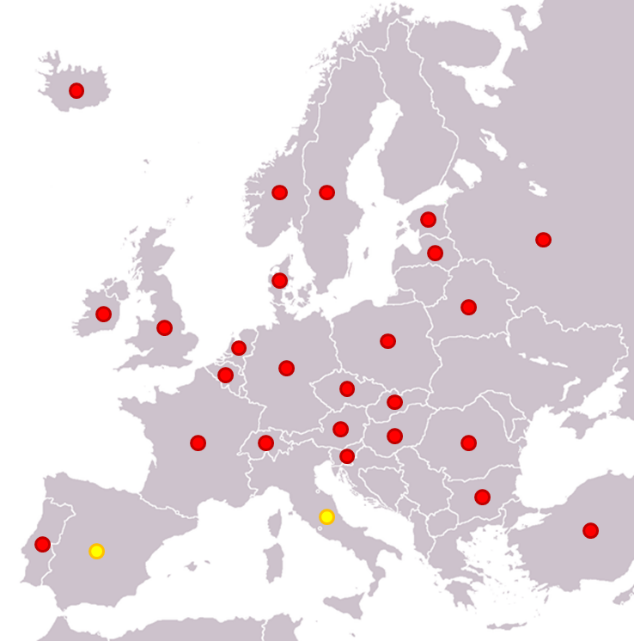

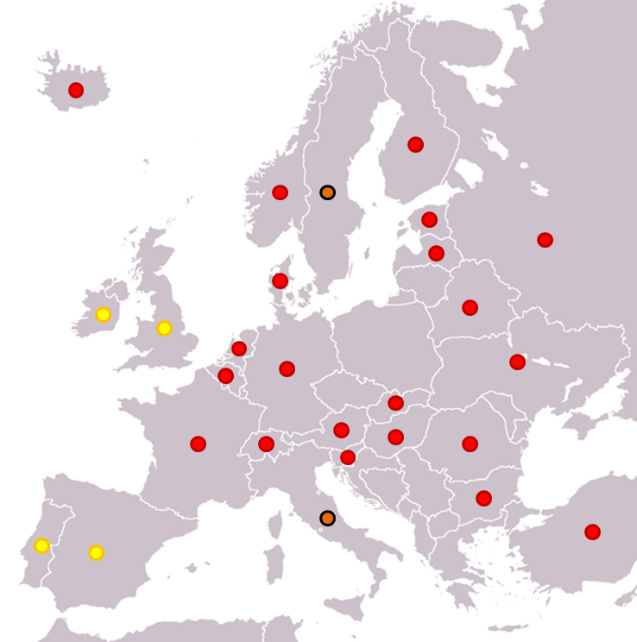


Eggs Internal tissues


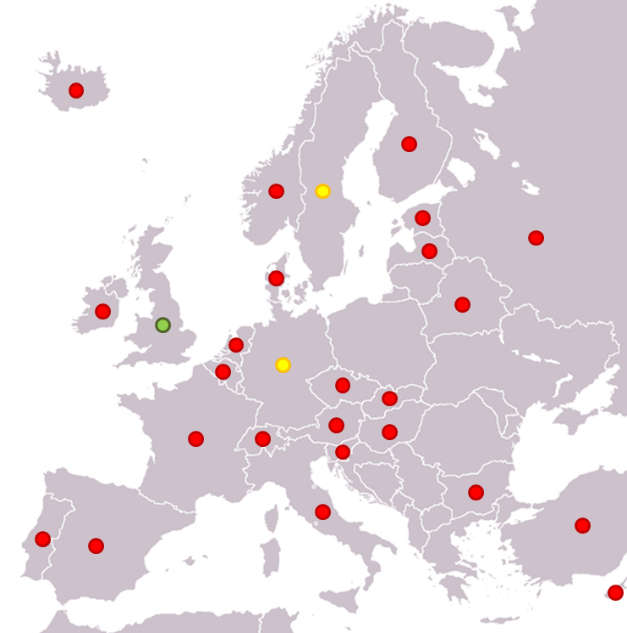

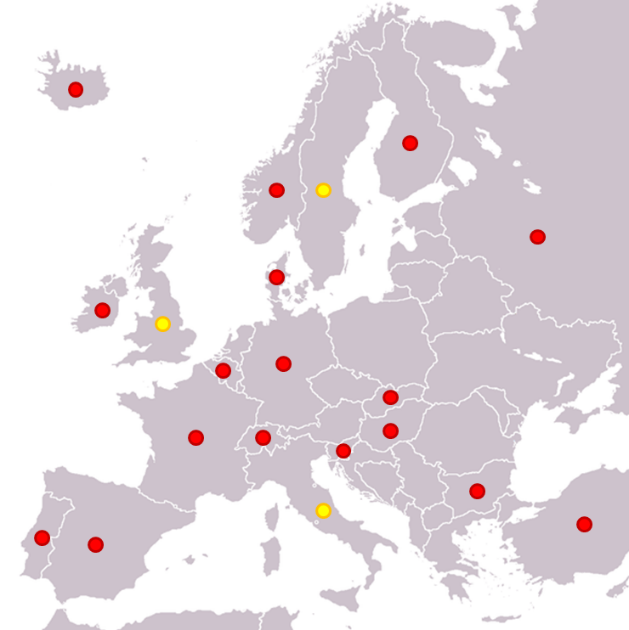


Pellets and food remains Preen oil


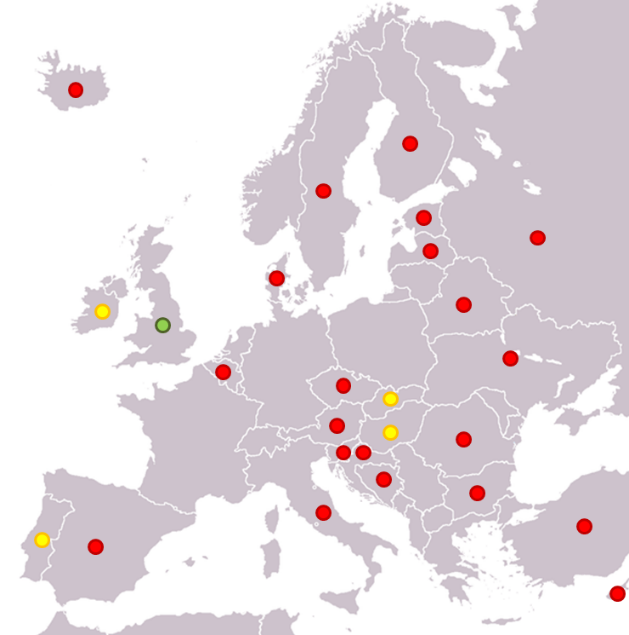

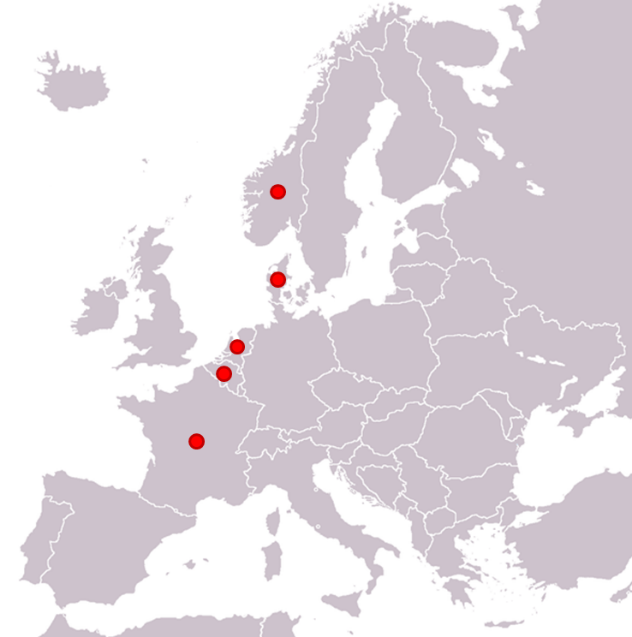


*Georgia (not shown in the maps) has one scheme that collect feathers and carcasses. Greenland (not shown in the maps) has two schemes that collect internal tissues, eggs, feathers, blood and preen oil. Luxembourg and Serbia sent questionnaires but their schemes do not collect samples (see Table 1).*


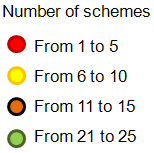


**S.I. Table 1.** Practical attributes of different sample types that are used in contaminant monitoring studies using raptors.

| **Matrix** | **Invasive technique that may require licensing to collect** | **Non invasive technique but legal issue** | **Storage** | | | | **Use of preservatives** | **Easy to find and collect** | **Useful for effect assessment** |
| --- | --- | --- | --- | --- | --- | --- | --- | --- | --- |
|  |  |  | **Room temperature** | **4°C** | **-20°C** | **-80°C** |  |  |  |
| Blood/serum/plasma | ✓ |  |  |  | ✓ | ✓ | ✓ |  | ✓ |
| Moulted feathers |  | ✓ | ✓ |  |  |  |  | ✓ | ✓ |
| Plucked feathers | ✓ |  | ✓ |  |  |  |  |  | ✓ |
| Addled or deserted eggs |  | ✓ |  |  | ✓ |  |  |  | ✓ |
| Internal tissues (carcasses) |  | ✓ |  |  | ✓ | ✓ |  |  | ✓ |
| Biopsies | ✓ |  |  |  | ✓ | ✓ |  |  | ✓ |
| Regurgitated Pellets |  | ✓ | ✓ |  |  |  |  | ✓ |  |
| Crop content (carcasses) |  | ✓ |  | ✓ | ✓ |  |  |  |  |
| Excrement collected by induced defecation | ✓* |  |  | ✓ | ✓ |  |  |  | ✓ |
| Excrement collected around roosts and nests |  | ✓* |  | ✓ | ✓ |  |  | ✓ | ✓ |
| Preen oil | ✓* | ✓* |  |  | ✓ |  |  |  |  |
| Clipped nails (carcasses) |  | ✓ | ✓ |  |  |  |  |  |  |

*(✓): Legal and/or ethical authorization may be required, specialised storage conditions and preservatives are required, easily found and/or collected, suitable for effect assessment.*Excrements/preen oil sampling may be considered both invasive and non-invasive. Faeces samples may be collected by inducing individuals to defecate on handling and preen oil can be collected from living birds by pressing the gland softly (invasive). However, faeces expelled and falling from nests can be picked up, and preen oil can be collected from carcasses (non-invasive).*

**S.I. Table 2.** Half-lives of some contaminants in blood compartments and internal tissues for different species.

| **Contaminant** | **Half-life (days)** | **Tissue** | **Species** | **References*** |
| --- | --- | --- | --- | --- |
| Brodifacoum (anticoagulant rodenticide) | 91.7 | Plasma | Mouse | (Vandenbroucke et al. 2008) |
|  | 307.4 | Liver | Mouse |  |
| Difenacoum (anticoagulant rodenticide) | 20.4 | Plasma | Mouse |  |
|  | 61.8 | Liver | Mouse |  |
| Warfarin (anticoagulant rodenticide) | 14.9 | Plasma | Mouse |  |
|  | 66.8 | Liver | Mouse |  |
| Perfluorooctane sulfonate (PFOS) | 6.86 | Serum | Mallard | (Newsted et al. 2006) |
|  | 17.5 | Liver | Mallard |  |
| Methylmercury (MeHg) | 14 | Blood | Monkey | (Rice 1989) |
|  | 56 | Brain | Monkey |  |
| Lead | 13 | Blood | California condor | (Fry and Maurer 2003) |
|  | Years | Bone | Birds | (Pain et al. 2005) |

*References

Fry DM, Maurer JR (2003) Assessment of lead contamination sources exposing California condors. Final Report. California Department of Fish and Game, Habitat Conservation Planning Branch, Sacramento, C.A, U.S.A.

Newsted JL, Beach SA, Gallagher SP, Giesy JP (2006) Pharmacokinetics and acute lethality of perfluorooctanesulfonate (PFOS) to juvenile mallard and northern bobwhite. Arch Environ Contam Toxicol 50:411–420. doi: 10.1007/s00244-005-1137-x

Pain DJ, Meharg AA, Ferrer M, et al (2005) Lead concentrations in bones and feathers of the globally threatened Spanish imperial eagle. Biological Conservation 121:603–610. doi: 10.1016/j.biocon.2004.06.012

Rice DC (1989) Brain and tissue levels of mercury after chronic methylmercury exposure in the monkey. J Toxicol Environ Health 27:189–198. doi: 10.1080/15287398909531290

Vandenbroucke V, Bousquet-Melou A, De Backer P, Croubels S (2008) Pharmacokinetics of eight anticoagulant rodenticides in mice after single oral administration. J Vet Pharmacol Ther 31:437–445. doi: 10.1111/j.1365-2885.2008.00979.x

**S.I. Table 3.** Pollutant group and type of raptor sample analyse by 249 European studies published between 1966 and 2015. Data were used to compile Table 2 in the main manuscript.

| **Pollutant type** | **Matrices** | **Reference** |
| --- | --- | --- |
| Cd | Feathers and Blood | *Martínez-López E., María-Mojica P., Martínez J.E., Calvo J.F., Romero D., García-Fernández A.J. 2005. Cadmium in feathers of adults and blood of nestlings of three raptor species from a nonpolluted Mediterranean forest, Southeastern Spain. Bull Environ Contam Toxicol 74:477-484.* |
| Cd | Liver | *Strong, R.J., Pereira, M.G., Shore, R.F., Henrys, P.A., Pottinger T,G. 2015. Feather corticosterone content in predatory birds in relation to body condition and hepatic metal concentration. Gen. Comp. Endocrinol.  14;214, 47-55.* |
| Cd | Liver, Kidney, Brain, Bone and Blood | *García-Fernández A.J., Sánchez-García J.A., Gómez-Zapata M., Luna A. 1996. Distribution of cadmium in blood and tissues of wild birds. Arch Environ Contam Toxicol 30:252-258.* |
| Hg | Feathers | *Berg W, Johnels AG, Sjöstrand B, Westermark T. 1966. Mercury content in Feathers of Swedish birds from the past 100 years. Oikos 17, 71–83.* |
| Hg | Feathers | *Broo, B., Odsjö, T. 1981. Mercury Levels in Feathers of Eagle-Owls Bubo bubo in a Captive, a Reintroduced and a Native Wild Population in SW Sweden. Holarctic Ecology  4 (4), 270-277.* |
| Hg | Feathers | *Dietz, R.; Riget, F. F.; Boertmann, D.; Sonne, C.; Olsen, M. T.; Fjeldsa, J.; Falk, K.; Kirkegaard, M.; Egevang, C.; Asmund, G.; Wille, F.; Moller, S., 2006. Time trends of mercury in feathers of West Greenland birds of prey during 1851-2003. Environmental Science & Technology 40, (19), 5911-5916.* |
| Hg | Feathers | *Lodenius M, Kuusela S. 1985. Mercury content in Feathers of the Kestrel (Falco tinnunculus L.) in Finland. Ornis Fenn 62, 158–160.* |
| Hg | Feathers | *Lourenço R, Tavares P, del Mar Delgado M, Rabaça J, Penteriani V. 2011. Superpredation increases mercury levels in a generalist top predator, the eagle owl. Ecotoxicology 20: 635-642.* |
| Hg | Feathers | *Martínez A, Crespo D, Fernández JÁ, Aboal JR, Carballeira A. 2012. Selection of flight feathers from Buteo buteo and Accipiter gentilis for use in biomonitoring heavy metal contamination. Sci Total Environ. 425:254-61.* |
| Hg | Feathers | *Odsjö T, Roos A, Johnels AG. 2004. The tail feathers of osprey nestlings (Pandion haliaetus L.) as indicators of change in mercury load in the environment of southern Sweden (1969-1998): a case study with a note on the simultaneous intake of selenium. Ambio. 33(3):133-7.* |
| Hg | Feathers | *Ortego, J., Jiménez, M., Díaz, M., Rodríguez, R.C., 2006, Mercury in Feathers of Nestling Eagle Owls, Bubo bubo L., and Muscle of their Main Prey Species in Toledo Province, Central Spain. Bulletin of Environmental Contamination and Toxicology 76, 648-655.* |
| Hg | Feathers | *Palma L, Beja P, Tavares PC, Monteiro LR. 2005. Spatial variation of mercury levels in nesting Bonelli’s eagles from Southwest Portugal: effects of diet composition and prey contamination. Environ Pollut.  134(3), 549-57.* |
| Hg | Feathers | *Scharenberg, W, Struwe-Juhl, B. 2000. Total Mercury in Feathers of White-Tailed Eagle (Haliaeetus albicilla L.) from Northern Germany over 50 Years. Bull. Environ. Contam. Toxicol. 64: 686-692.* |
| Hg | Feathers | *Solonen T, Lodenius M. 1984. Mercury in Finnish Sparrowhawks Accipiter nisus. Ornis Fennica 61, 58-63.* |
| Hg | Feathers | *Solonen T, Lodenius M. 1990. Feathers of birds of prey as indicators of mercury contamination in southern Finland. Holarct. Ecol. 13: 229-237.* |
| Hg | Feathers | *Weber, M., M. Niecke, K. Gedeon and H. Meyer. 2001. Mercury in the feathers of the Sparrowhawk (Accipiter nisus) from the Erz Mountains. Journal fur Ornithologie 142(3): 313-320.* |
| Hg | Feathers | *Westermark T, Odsjö T, Johnels AG. 1975. Mercury content of bird Feathers before and after Swedish ban on alkyl mercury in agriculture. Ambio 4, 87–92.* |
| Hg | Feathers and Blood | *Espín S, Martínez-López E, León-Ortega M, Calvo JF, García-Fernández, AJ. 2014a. Factors that influence mercury concentrations in nestling Eagle Owls (Bubo bubo). Sci Total Environ 470-471:1132–1139.* |
| Hg | Kidney | *Kitowski, I, Kowalski, R, Komosa, A, Lechowski, J, Grzywaczewski, G, Scibor, R, Pitucha, G, Chrapowicki, M. 2012. Diversity of total mercury concentrations in kidneys of birds from Eastern Poland. Ecology (Bratislava) 31, 12–21.* |
| Hg | Kidney | *Komosa, A, Kitowski, I, Kowalski, R, Pitucha, G, Komosa, Z, Grochowicz, J. 2009b. Total mercury concentration in kidneys of birds of prey from different part of Poland - some interspecies and geographical differences. Ecological Chemistry and Engineering S 16, S1.* |
| Hg | Liver and Kidney | *Falandysz J. 1986. Metals and organochlorines in adult qnd immature males of white-tailed eagle. Environmental Conservation 13, 69-79* |
| Hg | Liver and Kidney | *Henriksson, K., Karppanen, E. and Helminen, M. 1966. High residue levels of mercury in Finnish White-tailed eagles. - Ornis Fennica 43: 38-45.* |
| Hg | Liver and Kidney | *Holt, G., Frÿslie, A., Norheim, G. 1979. Mercury, DDE, and PCB in the avian fauna in Norway 1965-1976. Acta Veterinaria Scandinavica, Supplementum 70: 1-28* |
| Hg | Liver and Kidney | *Koeman J. H., Hadderingh R. H. and Bijleveld M. F. I. J. 1972. Persistent pollutants in the White tailed eagle in the Federal Republic of Germany. Biol. Conserv. 4, 373.* |
| Hg | Liver and Kidney | *Norheim, G., and Froslie, A. 1978. The degree of methylation and organ distribution of mercury in some birds of prey in Norway. Acta Pharmacol. Toxicol. 54, 196–204.* |
| Hg | Liver and Kidney | *Oehme, G. 1981. On the level of mercury residues in white-tailed eagles (Haliaeetus albicilla) found dead 1967-1978. Hercynia N F 18: 353-364.* |
| Hg | Liver, Kidney, Muscle, Brain | *Kalisińska, E, Gorecki, J, Lanocha, N, Okonska, A, Melgarejo, JB, Budis, H, Rzad, I, Golas, J. 2014. Total and Methylmercury in Soft Tissues of White-Tailed Eagle (Haliaeetus albicilla) and Osprey (Pandion haliaetus) Collected in Poland. AMBIO 43, 858–870.* |
| Hg | Liver, Kidney, Muscle, Brain, Feathers, Eggs | *Häkkinen, I., Häsänen, E. 1980. Mercury in eggs and nestlings of the osprey (Pandion haliaetus) in Finland and its bioaccumulation from fish. Annales Zoologici Fennici 17, 131–139.* |
| Hg | Muscle | *Dittmann, J, Altmeyer, M, Dmowski, K, Krüger, J, Müller, P, Wagner, G. 1990. Mercury concentrations in a white-tailed eagle (Haliaetus albicilla) from the vicinity of Warsaw, Poland. Environmental Conservation 17, 75–77.* |
| Hg, Cd and Pb | Blood | *Carneiro M, Colaço B, Brandão R, Azorín B, Nicolas O, Colaço J, Pires MJ, Agustí S, Casas-Díaz E, Lavin S, Oliveira PA. 2015. Assessment of the exposure to heavy metals in Griffon vultures (Gyps fulvus) from the Iberian Peninsula. Ecotoxicol Environ Saf 113: 295-301.* |
| Hg, Cd and Pb | Blood | *Espín S, Martínez-López E, Jiménez P, María-Mojica P, García-Fernández AJ. 2014c. Effects of heavy metals on biomarkers for oxidative stress in Griffon Vulture (Gyps fulvus). Environ Res 129:59–68.* |
| Hg, Cd and Pb | Blood | *Espín S, Martínez-López E, León-Ortega M, Martínez JE, García-Fernández AJ. 2014b. Oxidative stress biomarkers in Eurasian Eagle owls (Bubo bubo) in three different scenarios of heavy metal exposure. Environ Res 131:134–144.* |
| Hg, Cd and Pb | Blood, Liver and Kidney | *Carneiro M, Colaço B, Brandão R, Ferreira C, Santos N, Soeiro V, Colaço A, Pires MJ, Oliveira PA, Lavín S. 2014. Biomonitoring of heavy metals (Cd, Hg, and Pb) and metalloid (As) with the Portuguese common buzzard (Buteo buteo). Environ Monit Assess 186 (11): 7011-21.* |
| Hg, Cd and Pb | Feathers | *Altmeyer M., Dittmann J., Dmowski K., Wagner G., Müller P. 1991. Distribution of elements in flight feathers of a White-tailed Eagle. The science of the total environment 105, 157-164.* |
| Hg, Cd and Pb | Feathers | *Bustnes, J.O., Bårdsen, B-J, Bangjord, G., Lierhagen, S., Yoccoz, N. 2013a. Temporal trends (1986-2005) of essential and non-essential elements in a terrestrial raptor in northern Europe. Science of the total environment 458-460, 101-106.* |
| Hg, Cd and Pb | Feathers | *Dauwe T, Bervoets L, Pinxten R, Blust R, Eens M. 2003. Variation of heavy metals within and among feathers of birds of prey: effects of molt and external contamination. Environ Pollut. 124(3):429-36.* |
| Hg, Cd and Pb | Feathers, Liver and Kidney | *Castro I, Aboal JR, Fernandez JA, Carballeira A. 2011. Use of Raptors for Biomonitoring of Heavy Metals: Gender, Age and Tissue Selection. Bulletin of Environmental Contamination and Toxicology 86:347-351.* |
| Hg, Cd and Pb | Liver | *Hornfeldt B, Nyholm NEI. 1996. Breeding Performance of Tengmalm's Owl in a Heavy Metal Pollution Gradient. Journal of Applied Ecology 33(2): 377-386.* |
| Hg, Cd and Pb | Liver and Kidney | *Falandysz J., Ichihashi K., Szymczyk K., Yamasakis S., Mizera T. 2001. Metallic elements and metal poisoning among white-tailed sea eagles from the Baltic South coast. Marine Pollution Bulletin 42: 1190-1193* |
| Hg, Cd and Pb | Liver and Kidney | *Kenntner N, Tataruch F, Krone O. 2001. Heavy metals in soft tissue of white-tailed eagles found dead or moribund in Germany and Austria from 1993 to 2000. Environ Toxicol Chem. 20(8):1831-1837.* |
| Hg,Cd and Pb | Feathers | *Hahn E, Hahn K, Stoeppler M. 1993. Bird Feathers as bioindicators in areas of the German environmental specimen bank—bioaccumulation of mercury in food-chains and exogenous deposition of atmospheric pollution with lead and cadmium. Sci Total Environ 140, 259–270.* |
| Pb | Blood | *Czirjak G-A, Kenntner N, Krone O. 2010. Effect of blood lead levels on the constitutive immune response in white-tailed sea eagle (Haliaeetus albicilla) nestlings. Abstract book of the European Wildlife Disease Association Conference “Healthy wildlife, healthy people” Vlieland, The Netherlands, p. 91.* |
| Pb | Blood | *Espín, S., Martínez-López, E., Jiménez, P., María-Mojica, P., García-Fernández, AJ. 2015. Delta-aminolevulinic acid dehydratase (δALAD) activity in four free-living bird species exposed to different levels of lead under natural conditions. Environmental Research 137, 185-198.* |
| Pb | Blood | *García-Fernández A.J., Martínez-López E., Romero D., María-Mojica P., Godino A., Jiménez P. 2005a. High levels of blood lead in griffon vultures (Gyps fulvus) from Cazorla natural park (southern Spain). Environ Toxicol 20:459-463.* |
| Pb | Blood | *Gómez-Ramírez P., Martínez-López E., María-Mojica P., León-Ortega M., García-Fernández A.J. 2011. Blood lead levels and δ-ALAD inhibition in nestlings of Eurasian eagle owl (Bubo bubo) to assess lead exposure associated to an abandoned mining area. Ecotoxicology 20:131-138.* |
| Pb | Blood and Bone | *Gangoso L, Alvarez-Lloret P, Rodríguez-Navarro AA, Mateo R, Hiraldo F, Donázar JA. 2009. Long-term effects of lead poisoning on bone mineralization in vultures exposed to ammunition sources. Environ Pollut. 157(2):569-74.* |
| Pb | Blood and Pellets | *Pain, D.J., Carter, I., Sainsbury, A.W., Shore, R.F., Eden, P., Taggart, M.A., Konstantinos, S., Walker, L.A., Meharg, A.A., Raab, A., 2007, Lead contamination and associated disease in captive and reintroduced red kites Milvus milvus in England. Science of the Total Environment 376, 116-127.* |
| Pb | Blood, Liver and Bone | *Hernández, M., Margalida, A. 2009, Assessing the risk of lead exposure for the conservation of the endangered Pyrenean bearded vulture (Gypaetus barbatus) population. Environmental Research 109, 837-842.* |
| Pb | Blood, Liver and Bone | *Mateo, R, Estrada, J, Paquet, JY, Riera, X, Dominguez, L, Guitart, R, Martinez-Vilalta, A. 1999.Lead shot ingestion by marsh harriers Circus aeruginosus from the Ebro delta, Spain. Environmental Pollution 104, 435-440.* |
| Pb | Bone | *Komosa, A., Kitowski, I., 2008. Elevated lead concentration in skeletons of diurnal birds of prey Falconiformes and owls Strigiformes from eastern Poland. Ecological approach and review. Ecological Chemistry and Engineering 15 (3): 349-358* |
| Pb | Bone | *Mateo, R., Taggart, M., Meharg, A.A., 2003, Lead and arsenic in bones of birds of prey from Spain. Environmental Pollution 126, 107-114.* |
| Pb | Feathers | *Debén S, Angel Fernández J, Aboal JR, Carballeira A. 2012. Evaluation of different contour feather types for biomonitoring lead exposure in Northern goshawk (Accipiter gentilis) and tawny owl (Strix aluco). Ecotoxicol Environ Saf. 85:115-9.* |
| Pb | Feathers | *Martínez-López E., María-Mojica P., Martínez J.E., Peñalver J., Pulido M., Calvo J.F., García-Fernández A.J. 2004. Lead in feathers and δ-Aminolevulinic acid dehydratase activity in three raptor species from an unpolluted mediterranean forest (Southeastern Spain). Archiv Environ Contam Toxicol 47:270-275.* |
| Pb | Feathers and Bone | *Cardiel IE, Taggart MA, Mateo R. 2011. Using Pb-Al ratios to discriminate between internal and external deposition of Pb in feathers. Ecotoxicology and Environmental Safety 74: 911-917.* |
| Pb | Feathers and Bone | *Pain, D.J., Meharg, A.A., Ferrer, M., Taggart, M., Penteriani, V., 2005, Lead concentrations in bones and feathers of the globally threatened Spanish imperial eagle. Biological Conservation 121, 603-610.* |
| Pb | Feathers, Liver and Bone | *Rodriguez-Ramos Fernandez J, Höfle U, Mateo R, Nicolas de Francisco O, Abbott R, Acevedo P, Blanco JM. 2011. Assessment of lead exposure in Spanish imperial eagle (Aquila adalberti) from spent ammunition in central Spain. Ecotoxicology. 20(4):670-81.* |
| Pb | Liver | *Krone O, Langgemach T, Sömmer P, Kenntner N. 2003. Causes of mortality in white-tailed sea eagles from Germany. In: Sea Eagle 2000. Proc. Swedish Soc. For Nat. Conserv./SNF, Helander B, Marquiss M, Bowerman W (eds), Stockholm, Sweden, 211-218.* |
| Pb | Liver | *Mateo, R. Molina, R. Grífols, J. Guitart, R. 1997. Lead poisoning in a free ranging griffon vulture (Gyps fulvus). The Veterinary Record 140: 47-48.* |
| Pb | Liver | *Pain, D.J., Amiard-Triquet, C., 1993, Lead Poisoning of Raptors in France and Elsewhere. Ecotoxicology and Environmental Safety 25, 183-192.* |
| Pb | Liver | *Pain, D.J., Sears, J., Newton, I., 1995, Lead concentrations in birds of prey in Britain. Environmental Pollution 87, 173-180.* |
| Pb | Liver and Kidney | [*Krone O, Kenntner N, Trinogga A, Nadjafzadeh N, Scholz F, Sulawa J, Totschek K, Schuck-Wersig P and Zieschank R. 2009b. Lead poisoning in white-tailed sea eagles: Causes and approaches to solutions in Germany. In Watson R T, Fuller M, Pokras A and Hunt W G (eds.) Ingestion of lead from spent ammunition: implications for wildlife and humans. The Peregrine Fund, Boise, Idaho, USA, 289-301. http://www.peregrinefund.org/Lead_conference/PDF/0207%20Krone.pdf*](http://www.peregrinefund.org/Lead_conference/PDF/0207%20Krone.pdf) |
| Pb | Liver, Kidney | *Helander B, Axelsson J, Borg H, Holm K, Bignert A. 2009. Ingestion of lead from ammunition and lead concentrations in white-tailed sea eagles (Haliaeetus albicilla) in Sweden. Sci Total Environ. 407(21):5555-63.* |
| Pb | Liver, Kidney, Brain and Bone | *Garcia-Fernandez, A.J., Romero, D., Martinez-Lopez, E., Navas, I., Pulido, M., Maria-Mojica, P., 2005b, Environmental lead exposure in the European kestrel (Falco tinnunculus) from southeastern Spain: the influence of leaded gasoline regulations. Bull Environ Contam Toxicol 74, 314-319.* |
| Pb | Liver, Kidney, Brain, Bone and Blood | *García-Fernández, A.J., Motas-Guzmán, M., Navas, I., María-Mojica, P., Luna, A., Sánchez-García, J.A., 1997, Environmental Exposure and Distribution of Lead in Four Species of Raptors in Southeastern Spain. Archives of Environmental Contamination and Toxicology 33, 76-82.* |
| Pb | Pellet | *Mateo, R., Cadenas, R., Máñez, M., Guitart, R. 2001. Lead shot ingestion in two raptor species from Doñana, Spain. Ecotoxicology and Environmental Safety 48: 6-10.* |
| Pb | Pellet | *Mateo, R., Green, A.J., Lefranc, H., Baos, R., Figuerola, J. 2007. Lead poisoning in wild birds from southern Spain: a comparative study of wetland areas and species affected, and trends over time. Ecotoxicology and Environmental Safety 66: 119-126.* |
| Pb and Cd | Blood | *Baos R, Jovani R, Forero MG, Tella JL, Gómez G, Jiménez B, González MJ, Hiraldo F. 2006a. Relationships between T-cell-mediated immune response and Pb, Zn, Cu, Cd, and As concentrations in blood of nestling white storks (Ciconia ciconia) and black kites (Milvus migrans) from Doñana (southwestern Spain) after the Aznalcóllar toxic spill. Environmental Toxicology and Chemistry 25: 1153-1159.* |
| Pb and Cd | Blood | *Baos R, Jovani R, Pastor N, Tella JL, Jiménez B, Gómez G, González MJ, Hiraldo F. 2006b. Evaluation of Genotoxic effects of heavy metals and arsenic in wild nestling white storks (Ciconia ciconia) and black kites (Milvus migrans) from southwestern Spain after a mining accident. Environmental Toxicology and Chemistry 25: 2794-2803.* |
| Pb and Cd | Blood | *Benito V, Devesa V, Muñoz O, Suñer MA, Montoro R, Baos R, et al. 1999. Trace elements in blood collected from birds feeding in the area around Doñana National Park affected by the toxic spill from the Aznalcóllar mine. The Science of the Total Environment 242(1-3): 309-323.* |
| Pb and Cd | Blood | *Blanco G1, Jiménez B, Frías O, Millan J, Dávila JA. 2004. Contamination with nonessential metals from a solid-waste incinerator correlates with nutritional and immunological stress in prefledgling black kites (Milvus migrans). Environ Res, 94(1), 94-101.* |
| Pb and Cd | Bone | *Komosa, A., Kitowski, I., Chibowski, S., Solecki, J., Orzeł, J., Różański, P., 2009a. Selected radionuclides and heavy metals in skeletons of birds of prey from eastern Poland. Journal of Radioanalytical and Nuclear Chemistry 281, 467-478.* |
| Pb and Cd | Eggs | *Ramón, L. 2009. Metales pesados en cáscaras de huevos de las poblaciones de aguilucho cenizo (Circus pygargus) del sur de España. Rev Toxicología 26:58* |
| Pb and Cd | Eggs and Blood | *Blanco G, Frías O, Jiménez B, Gómez G. 2003. Factors influencing variability and potential uptake routes of heavy metals in black kites exposed to emissions from a solid-waste incinerator. Environmental Toxicology and Chemistry 22: 2711-2718.* |
| Pb and Cd | Feathers | *Denneman WD, Douben PET. 1993. Trace metals in primary feathers of the Barn Owl (Tyto alba guttatus) in The Netherlands. Environmental Pollution 82(3): 301-310.* |
| Pb and Cd | Feathers | *Solonen, T., M. Lodenius and E. Tulisalo. 1999. Metal levels of feathers in birds of various food chains in southern Finland. Ornis Fennica 76(1): 25-32* |
| Pb and Cd | Feathers, Egg, Blood, Liver and Kidney | *Ek KH, Morrison GM, Lindberg P, Rauch S. 2004. Comparative tissue distribution of metals in birds in Sweden using ICP-MS and laser ablation ICP-MS. Arch Environ Contam Toxicol. 47(2), 259-69.* |
| Pb and Cd | Feathers, Liver, Kidney and Muscle | *Naccari C, Cristani M, Cimino F, Arcoraci T, Trombetta D. 2009. Common buzzards (Buteo buteo) bioindicators of heavy metals pollution in Sicily (Italy). Environ Int 35(3):594-8.* |
| Pb and Cd | Liver | *Pérez-López, M., Hermoso de Mendoza, M., López Beceiro, A., Soler Rodríguez, F., 2008, Heavy metal (Cd, Pb, Zn) and metalloid (As) content in raptor species from Galicia (NW Spain). Ecotoxicology and Environmental Safety 70, 154-162.* |
| Pb and Cd | Liver and Brain | *Zaccaroni, A., Amorena, M., Naso, B., Castellani, G., Lucisano, A., Stracciari, G.L., 2003, Cadmium, chromium and lead contamination of Athene noctua, the little owl, of Bologna and Parma, Italy. Chemosphere 52, 1251-1258.* |
| Pb and Cd | Liver, Bone, Kidney, Muscle and Feathers | *Battaglia A, Ghidini S, Campanini G, Spaggiari R. 2005. Heavy metal contamination in little owl (Athene noctua) and common buzzard (Buteo buteo) from northern Italy. Ecotoxicology and Environmental Safety 60(1): 61-66.* |
| Pb and Cd | Liver, Kidney and Bone | *Esselink H., van der Geld, F. M.; Jager, L. P.; Posthuma-Trumpie, G. A.; Zoun, P. E. F.; Baars, A. J. 1995. Biomonitoring heavy metals using the barn owl (Tyto alba guttata): sources of variation especially relating to body condition. Arch Environ Contam Toxicol. 28, 471-486.* |
| Pb and Cd | Liver, Kidney and Bone | *Hontelez L.C.M.P., van den Dungen H.M., Baars A.J., 1992. Lead and cadmium in birs in the Netherlands – a preliminary study. Archives of Environmental Contamination and Toxicology 23(4): 453-456.* |
| Pb and Cd | Liver, Kidney and Bone | *Jager, L.P., Rijnierse, F.V.J., Esselink, H., Baars, A.J., 1996, Biomonitoring with the Buzzard (Buteo buteo) in the Netherlands: Heavy metals and sources of variation. Journal of Ornithology 137, 295-318.* |
| Pb and Cd | Liver, Kidney, Brain and Muscle | *Kalisińska, E, Salicki, W, Jackowski, A. 2006. Six Trace Metals in White-Tailed Eagle from Northwestern Poland. Polish J. of Environ. Stud. 15 (5), 727-737.* |
| Pb and Cd | Liver, Kidney, Brain, Bone and Blood | *Garcia-Fernandez, A.J., Sanchez-Garcia, J.A., Jimenez-Montalban, P., Luna, A., 1995, Lead and cadmium in wild birds in southeastern Spain. Environmental Toxicology and Chemistry 14, 2049-2058.* |
| Pb and Hg | Liver and Kidney | *Krone O, Kenntner N, Tataruch F. 2009a. Gefährdungsursachen des Seeadlers (Haliaeetus albicilla L. 1758). Denisia 27: 139-146.* |
| PFASs | Eggs | *Holmström KE, Johansson AK, Bignert A, Lindberg P, Berger U. 2010. Temporal trends of perfluorinated surfactants in Swedish peregrine falcon Eggs (Falco peregrinus), 1974-2007. Environ Sci Technol 44(11):4083-8.* |
| PFASs | Eggs | *Ahrens L, Herzke D, Huber S, Bustnes JO, Bangjord G, Ebinghaus R. 2011. Temporal trends and pattern of polyfluoroalkyl compounds in Tawny Owl (Strix aluco) eggs from Norway, 1986-2009. Environ Sci Technol. 45(19), 8090-7.* |
| PFASs | Feathers and Liver | *Meyer, J., Jaspers, V.L., Eens, M., de Coen, W., 2009. The relationship between perfluorinated chemical levels in the feathers and livers of birds from different trophic levels. Sci Total Environ 407, 5894-5900.* |
| PFASs | Feathers and Preen oil | *Herzke, D., Jaspers, V.L.B., Boertmann, D., Rasmussen, L.M., Sonne, C., Dietz, R., Covaci, A., Eens, M., Bustnes, J.O., 2011. PFCs in feathers of white-tailed eagles (Haliaeetus albicilla) from Greenland and Norway, useful for nondestructive sampling? Organohalogen Compounds 73, 1337e1339.* |
| PFASs | Feathers, Liver, Muscle, Fat and Preen oil | *Jaspers VL, Herzke D, Eulaers I, Gillespie BW, Eens M. 2013a. Perfluoroalkyl substances in soft tissues and tail feathers of Belgian barn owls (Tyto alba) using statistical methods for left-censored data to handle non-detects. Environ Int. 52:9-16..* |
| PFASs | Liver | *Kannan K, Corsolini S, Falandysz J, Oehme G, Focardi S, Giesy JP.2002. Perfluorooctanesulfonate and related fluorinated hydrocarbons in marine mammals, fishes, and birds from coasts of the Baltic and the Mediterranean Seas. Environ Sci Technol. 36(15):3210-6.* |
| PFASs and POPs | Eggs | *Faxneld S, Helander B, Bäcklin B-M, Moraeus C, Roos A, Berger U, Egebäck A-L, Strid A,, Kierkegaard A, Bignert B. 2014. Biological effects and environmental contaminants in herring and Baltic Sea top predators.Swedish Museum of Natural History, Rapport nr 6:2014* |
| PFASs and POPs | Eggs and Feathers | *Gjershaug, J. O.; Kalas, J. A.; Nygard, T.; Herzke, D.; Folkestad, A. O., 2008. Monitoring of raptors and their contamination levels in Norway. Ambio 37, (6), 420-424.* |
| PFASs and POPs | Plasma | *Bustnes JO, Bårdsen BJ, Herzke D, Johnsen TV, Eulaers I, Ballesteros M, Hanssen SA, Covaci A, Jaspers VL, Eens M, Sonne C, Halley D, Moum T, Nøst TH,Erikstad KE, Ims RA. 2013b. Plasma concentrations of organohalogenated pollutants in predatory bird nestlings: associations to growth rate and dietary tracers. Environ Toxicol Chem. 32(11):2520-7.* |
| PFASs and POPs | Plasma | *Sonne C, Bustnes JO, Herzke D, Jaspers VLB, Covaci A, Halley DJ, et al. 2010. Relationships between organohalogen contaminants and blood plasma clinical-chemical parameters in chicks of three raptor species from Northern Norway. Ecotoxicology and Environmental Safety 73(1): 7-17.* |
| PFASs and POPs | Plasma | *Sonne C, Bustnes JO, Herzke D, Jaspers VLB, Covaci A, Halley DJ, Moum T, Eulaers I, Eens M, Ims RA, Hanssen SA, Erikstad KE, Johnsen TV, Schnug L, Rigét FF, Jensen AL, Kjelgaard-Hansen M. 2012. Blood plasma clinical-chemical parameters as biomarker endpoints for organohalogen-contaminant exposure in Norwegian raptor nestlings. Ecotoxicology and Environmental Safety 80, 76-83.* |
| Pharmaceuticals | Liver and Kidney | *Zorrilla, I., Martinez, R., Taggart, M.A., Richards, N. 2015. Suspected flunixin poisoning of a wild Eurasian Griffon Vulture from Spain. Conserv Biol.  29(2), 587-92.* |
| Pharmaceuticals | Plasma | *García-Fernández, A.J., María-Mojica, P., Grau, L.V., Jiménez, P., Martínez-López, E., Navas, I., 2013. Residuos de antibióticos de uso veterinario en plasma sanguíneo de una población de buitre leonado (gyps fulvus). Rev Toxicol 30 (1).* |
| POPs | Blood | *Goutner V, Skartsi T, Konstantinou IK, Sakellarides TM, Albanis TA, Vasilakis D, Elorriaga J, Poirazidis K. 2011. Organochlorine residues in Blood of cinereous vultures and Eurasian griffon vultures in a northeastern Mediterranean area of nature conservation. Environ Monit Assess 183(1-4), 259-71.* |
| POPs | Blood | *Martínez-López E., Romero D., María-Mojica P., Martínez J.E., Calvo J.F., García-Fernández A.J. 2009. Changes in blood pesticide levels in Booted eagle (Hieraaetus pennatus) associated with agricultural land practices. Ecotoxicol Environ Saf 72:45-50.* |
| POPs | Blood | *Movalli P, Lo Valvo M, Pereira MG, Osborn D. 2008. Organochlorine pesticides and polychlorinated biphenyl congeners in lanner Falco biarmicus feldeggli Schlegel chicks and lanner prey in Sicily, Italy. Ambio.37(6):445-51.* |
| POPs | Blood | *Navas, I., Martínez-López, E., Romero, D., Molina, I., María-Mojica, P. and García- Fernández, A.J. 2005b. Lindane and endosulfan blood concentrations in healthy nestlings of Spanish imperial eagle and Bonelli’s eagle: relationships with infectious and parasitic pathological processes. Rev. Toxicol. 22, 112. (In Spanish).* |
| POPs | Blood | *Olsson, A., Ceder, K., Bergman, Å., Helander, B., 2000, Nestling Blood of the White-Tailed Sea Eagle (Haliaeetus albicilla) as an Indicator of Territorial Exposure to Organohalogen Compounds−An Evaluation. Environmental Science & Technology 34, 2733-2740.* |
| POPs | Blood, Liver and Brain | *Gómez-Ramírez, P., Martínez-López, E., Hernández-García, A., María-Mojica, P., Botella, F., Sánchez, A., Martínez, J.E., Calvo, J.F., García-Fernández A.J 2005. Biomonitoring of organochlorine insecticides in blood and tissues of Eagle owl (Bubo bubo) from Alicante and Murcia (Spain). Rev. Toxicol. 22, 140. (In Spanish).* |
| POPs | Blood, Liver, Fat, Kidney, Muscle, Preen oil | *Jaspers VL, C. Sonne, F. Soler-Rodriguez, D. Boertmann, R. Dietz, M. Eens, L.M. Rasmussen, A. Covaci. 2013b. Persistent organic pollutants and methoxylated polybrominated diphenyl ethers in different tissues of white-tailed eagles (Haliaeetus albicilla) from West Greenland. Environmental Pollution 175, 137e146.* |
| POPs | Brain and Fat | *Naert C, Van Peteghem C, Kupper J, Jenni L, Naegeli H. 2007. Distribution of polychlorinated biphenyls and polybrominated diphenyl ethers in birds of prey from Switzerland. Chemosphere 68(5), 977-87.* |
| POPs | Eggs | *Bustnes JO, Yoccoz NG, Bangjord G, Herzke D, Ahrens L, Skaare JU. 2011. Impacts of climate and feeding conditions on the annual accumulation (1986-2009) of persistent organic pollutants in a terrestrial raptor. Environ Sci Technol. 45 (17): 7542-7.* |
| POPs | Eggs | *Bustnes, J. O.; Yoccoz, N. G.; Bangjord, G.; Polder, A.; Skaare, J. U., 2007. Temporal trends (1986-2004) of organochlorines and brominated flame retardants in tawny owl eggs from northern Europe. Environmental Science & Technology 41, (24), 8491-8497.* |
| POPs | Eggs | *Bustnes, J.O., Bangjord, G., Yoccoz, N.G. 2015. Variation in concentrations of organochlorines and brominated flame retardants among eggs in abandoned clutches of a terrestrial raptor. Chemosphere 118, 357-60.* |
| POPs | Eggs | *Conrad B. 1977. Die Giftbelastung der Vogelwelt Deutschlands. Vogelkundliche Bibliothek Vol. 5, Kilda-Verlag, Greven* |
| POPs | Eggs | *Crosse, J.D., Shore, R.F., Wadsworth, R.A., Jones, K.C., Pereira, M.G. 2012. Long-term trends in PBDEs in sparrowhawk (Accipiter nisus) eggs indicate sustained contamination of UK terrestrial ecosystems. Environ Sci Technol 46 (24): 13504-11.* |
| POPs | Eggs | *Dell'Omo G, Costantini D, Wright J, Casagrande S, Shore RF. 2008. PCBs in the Eggs of Eurasian Kestrels Indicate Exposure to Local Pollution. AMBIO: A Journal of the Human Environment 37: 452-456.* |
| POPs | Eggs | *Gómara, B., Fernández, M.A., Baos, R., Herrero, L., Jiménez, B., Abad, E., Hiraldo, F., Ferrer, M., Rivera, J., González, M.J. 2002. Presence of organochlorine pollutants (PCDDs, PCDFs, PCBs and DDTs) in eggs of predatory birds from Doñana National Park, Spain. Organohalogen Compounds, 58: 441-444.* |
| POPs | Eggs | *Gómara, B., González, M.J., 2006, Enantiomeric fractions and congener specific determination of polychlorinated biphenyls in eggs of predatory birds from Doñana National Park (Spain). Chemosphere 63, 662-669.* |
| POPs | Eggs | *Gómara, B., González, M.J., Baos, R., Hiraldo, F., Abad, E., Rivera, J., Jiménez, B., 2008, Unexpected high PCB and total DDT levels in the breeding population of red kite (Milvus milvus) from Doñana National Park, south-western Spain. Environment International 34, 73-78.* |
| POPs | Eggs | *Gómez-Ramírez, P., Martínez-López, E., García-Fernández, A.J., Zweers, A.J., and Van den Brink, N.W. 2012a. Organohalogen exposure in a Eurasian Eagle owl (Bubo bubo) population from Southeastern Spain: temporal-spatial trends and risk assessment. Chemosphere 88, 903–911.* |
| POPs | Eggs | *Guerra P, Alaee M, Jiménez B, Pacepavicius G, Marvin C, MacInnis G, Eljarrat E, Barceló D, Champoux L, Fernie K.2012. Emerging and historical brominated flame retardants in peregrine falcon (Falco peregrinus) Eggs from Canada and Spain. Environ Int 40, 179-86.* |
| POPs | Eggs | *Helander B, 1994. Productivity in relation to residue levels of DDE in the eggs of White-tailed sea eagles Haliaeetus albicilla in Sweden. Pp. 735-738 in: Meyburg, B.-U. & Chancellor, R.D. (eds.), Raptor Conservation Today.WWGBP/The Pica Press.* |
| POPs | Eggs | *Helander B, Bignert A, Asplund L, 2008. Using raptors as environmental sentinels: monitoring the white-tailed sea eagle Haliaeetus albicilla in Sweden. Ambio 37(6):425-31.* |
| POPs | Eggs | *Helander B, Olsson A, Bignert A, Asplund L, Litzén K. 2002. The role of DDE, PCB, coplanar PCB and eggshell parameters for reproduction in the white-tailed sea eagle (Haliaeetus albicilla) in Sweden. Ambio. 31(5):386-403.* |
| POPs | Eggs | *Henny CJ, Galushin VM, Khokhlov AN, Malovichko LV, Iljukh MP. 2003. Organochlorine Pesticides in Eggs of Birds of Prey from the Stavropol Region, Russia. Bulletin of Environmental Contamination and Toxicology 71(1): 163-169.* |
| POPs | Eggs | *Hernández, M., González, L.M., Oria, J., Sánchez, R., Arroyo, B., 2008, Influence of contamination by organochlorine pesticides and polychlorinated biphenyls on the breeding of the Spanish Imperial Eagle (Aquila adalberti). Environmental Toxicology and Chemistry 27, 433-441.* |
| POPs | Eggs | *Herzke, D., Berger, U., Kallenborn, R., Nygård, T., Vetter, W., 2005, Brominated flame retardants and other organobromines in Norwegian predatory bird eggs. Chemosphere 61, 441-449.* |
| POPs | Eggs | *Herzke, D., Kallenborn, R., Nygård, T., 2002, Organochlorines in egg samples from Norwegian birds of prey: Congener-, isomer- and enantiomer specific considerations. Science of the Total Environment 291, 59-71.* |
| POPs | Eggs | *Isaksson A, Helander B., 2003. Organochlorines in the laying sequence of the White-tailed Sea Eagle and Black Guillemot - a preliminary study. In: SEA EAGLE 2000, pp. 257-264.* |
| POPs | Eggs | *Janák K, Sellström U, Johansson A-K, Becher G, de Wit CA, Lindberg P, Helander B, 2008. Enantiomer-specific accumulation of hexabromocyclododecanes in eggs of predatory birds. Chemosphere 73:S193–S200.* |
| POPs | Eggs | *Jaspers V, Covaci A, Maervoet J, Dauwe T, Voorspoels S, Schepens P, Eens M. 2005. Brominated flame retardants and organochlorine pollutants in eggs of little owls (Athene noctua) from Belgium. Environmental Pollution 136, 81-88.* |
| POPs | Eggs | *Jensen S, Helander B, 2003. Retrospective determination of DDT and PCB substances in White-tailed Sea Eagles back to 1934 using fat remaining in blown eggs – a preliminary study. In: SEA EAGLE 2000, pp. 269-275.* |
| POPs | Eggs | *Jiménez, B., Gómara, B., Baos, R., Hiraldo, F., Eljarrat, E., Rivera, J. and González, M.J. 2000. A study of the toxic equivalents derived from PCDDs, PCDFs and dioxinlike PCBs in two bird species (Ciconia ciconia and Milvus migrans) nesting in a protected area (Doñana National Park, Spain). Organohalogen Compd. 46, 542–545.* |
| POPs | Eggs | *Jiménez, B., Merino, R., Abad, E., Rivera, J., Olie, K., 2007, Evaluation of organochlorine compounds (PCDDs, PCDFs, PCBs and DDTs) in two raptor species inhabiting a Mediterranean island in Spain. Environmental Science and Pollution Research 14, 61-68.* |
| POPs | Eggs | *Jiménez, B., Merino, R., Olie, K., Blanco, G. & Frias, O. 2004. Biomonitoring of organochlorine compounds (PCDDs, PCDFs, PCBs and DDTs) near a municipal solid waste incinerator using black kites (Milvus migrans) as sentinel organism. Organohalogen Compounds, 66: 1877-1881* |
| POPs | Eggs | *Jimenez, B., Merino, R., Olie, K., Blanco, G. and Frias, O. 2002. Biomonitoring of organochlorine compounds (PCDDs, PCDFs, PCBs and DDTs) near a municipal solid waste incinerator using black kites (Milvus migrans) as sentinel organism. Organohalogen Compd. 66, 1877–1881.* |
| POPs | Eggs | *Jiménez, B., Rodríguez-Estrella, R., Merino, R., Gómez, G., Rivera, L., José González, M., Abad, E., Rivera, J., 2005, Results and evaluation of the first study of organochlorine contaminants (PCDDs, PCDFs, PCBs and DDTs), heavy metals and metalloids in birds from Baja California, México. Environmental Pollution 133, 139-146.* |
| POPs | Eggs | *Johansson, A. K.; Sellstrom, U.; Lindberg, P.; Bignert, A.; de Wit, C. A., 2011. Temporal trends of polybrominated diphenyl ethers and hexabromocyclododecane in Swedish Peregrine Falcon (Falco peregrinus peregrinus) eggs. Environment international 37, (4), 678-686.* |
| POPs | Eggs | *Johansson, A.K., Sellström, U., Lindberg, P., Bignert, A., de Wit, C.A., 2009, Polybrominated diphenyl ether congener patterns, hexabromocyclododecane, and brominated biphenyl 153 in eggs of peregrine falcons (Falco peregrinus) breeding in Sweden. Environmental Toxicology and Chemistry 28, 9-17.* |
| POPs | Eggs | *Kubistova I, Vavrova M, Literak I. 2003. Polychlorinated biphenyls in raptor and owl eggs in the Czech Republic. Vet Med 48: 363-368.* |
| POPs | Eggs | *Lindberg P, Sellström U, Häggberg L, de Wit CA. 2004. Higher Brominated Diphenyl Ethers and Hexabromocyclododecane Found in Eggs of Peregrine Falcons (Falco peregrinus) Breeding in Sweden. Environmental Science & Technology 38(1): 93-96.* |
| POPs | Eggs | *Malisch R, Baum F. 2007. PCDD/Fs, dioxin-like PCBs and marker PCBs in Eggs of peregrine falcons from Germany. Chemosphere 67(9), S1-15.* |
| POPs | Eggs | *Mañosa, S., Mateo, R., Freixa, C., Guitart, R., 2003, Persistent organochlorine contaminants in eggs of northern goshawk and Eurasian buzzard from northeastern Spain: temporal trends related to changes in the diet. Environmental Pollution 122, 351-359.* |
| POPs | Eggs | *Martínez-López E., María-Mojica P., Martínez J.E., Calvo J.F., Wright J., Shore R.F., Romero D., García-Fernández A.J. 2007. Organochlorine residues in Booted eagle (Hieraaetus pennatus) and Goshawk (Accipiter gentilis) eggs from Southeastern Spain. Environ Toxicol Chem 26:2373-2378.* |
| POPs | Eggs | *Mateo, R., Carrillo, J., Guitart, R., 2000, p,p′-DDE Residues in Eggs of European Kestrel Falco tinnunculus from Tenerife, Canary Islands, Spain. Bulletin of Environmental Contamination and Toxicology 65, 780-785.* |
| POPs | Eggs | *Merino R, Abad E, Rivera J, Olie K. 2007. Evaluation of Organochlorine Compounds (PCDDs, PCDFs, PCBs and DDTs) in Two Raptor Species Inhabiting a Mediterranean Island in Spain (8 pp). Environ Sci Pollut Res Int. Suppl 1:61-8.* |
| POPs | Eggs | *Merino, R., Blanco, G., Abad, E., Rivera, J. & Jiménez, B. 2002. Toxicity derived from PCDDs, PCDFs, and dioxin-like PCBs in black kites (Milvus migrans) nesting near a municipal solid waste incinerator. Preliminary results: population effects. Organohalogen Compounds, 57: 435-438.* |
| POPs | Eggs | *Merino, R., Bordajandi, L.R., Abad, E., Rivera, J. and Jiménez, B. 2005. Evaluation of organochlorine compounds in peregrine falcon (Falco peregrinus) and their main prey (Columba livia) inhabiting central Spain. Environ. Toxicol. Chem. 24, 2088–2093.* |
| POPs | Eggs | *Newton, I., Bogan, J., 1978, The Role of Different Organo-Chlorine Compounds in the Breeding of British Sparrowhawks. Journal of Applied Ecology 15, 105-116.* |
| POPs | Eggs | *Nordlöf U, Helander B, Bignert A, Asplund L, 2010. Levels of brominated flame retardants and methoxylated polybrominated diphenyl ethers in eggs of white-tailed sea eagles breeding in different regions of Sweden. Sci. Tot. Env. 409:238–246.* |
| POPs | Eggs | *Nordlöf U, Helander B, Eriksson U, Zebühr Y, Asplund L, 2012a. Comparison of organohalogen compounds in a White-tailed sea eagle egg laid in 1941 with five eggs from 1996-2001. Chemosphere 88:286-291.* |
| POPs | Eggs | *Nordlöf U, Helander B, Zebühr Y, Bignert A, Asplund L, 2012b. Polychlorinated dibenzo-p-dioxins, polychlorinated dibenzofurans and non-ortho-PCBs in eggs of white-tailed sea eagles collected along the Swedish coast in the Baltic Sea. Sci. Tot. Env. 438:166–173.* |
| POPs | Eggs | *Nygåard, T., 1983. Pesticide-residues and shell thinning in eggs of peregrines in Norway. Ornis Scandinavica 14, (2), 161-166.* |
| POPs | Eggs | *Olsson A, Asplund L, Helander B, Bergman Å, Kylin H, 1993. Isomer specific analysis of PCB and PCB methyl sulphones in eggs from white-tailed sea eagle. Organohalogen Compounds 14:113-116.* |
| POPs | Eggs | *Pereira, M.G., Walker, L.A., Wright, J., Best, J., Shore, R.F.  2009. Concentrations of polycyclic aromatic hydrocarbons (PAHs) in the eggs of predatory birds in Britain.  Environmental Science & Technology 43, 9010-9015.* |
| POPs | Eggs | *Provini A, Galassi S. 1999. Polychlorinated Biphenyls and Chlorinated Pesticides in Bird Eggs from Calabria (Southern Italy). Ecotoxicology and Environmental Safety 43(1): 91-97.* |
| POPs | Eggs | *Roos A, Bäcklin B-M, Helander B, Rigét F, Eriksson U, 2012. Improved reproductive success in otters (Lutra lutra), grey seals (Halichoerus grypus) and sea eagles (Haliaeetus albicilla) from Sweden in relation to concentrations of organochlorine contaminants. Env. Pollut. 170:268-275.* |
| POPs | Eggs | *Scharenberg W, Struwe-Juhl B. 2006. White-tailed eagles (Haliaeetus albicilla) in Schleswig-Holstein no longer endangered by organochlorines. Bull Environ Contam Toxicol 77(6), 888-95.* |
| POPs | Eggs | *Scharenberg, W, Looft, V. 2004. Reduction of Organochlorine Residues in Goshawk Eggs (Accipiter gentilis) from Northern Germany (1971-2002) and Increasing Eggshell Index. Ambio, 33 (8), 495-498.* |
| POPs | Eggs | *Sellström U, Jansson B, Kierkegaard A, de Wit C, Odsjö T, Olsson M. 1993. Polybrominated diphenyl ethers (PBDE) in biological samples from the Swedish environment. Chemosphere 26(9): 1703-1718.* |
| POPs | Eggs | *Vetter, W., von der Recke, R., Herzke, D., Nygård, T., 2008, Detailed analysis of polybrominated biphenyl congeners in bird eggs from Norway. Environmental Pollution 156, 1204-1210.* |
| POPs | Eggs | *Vorkamp K, Møller S, Falk K, Rigét FF, Thomsen M, Sørensen PB. 2014. Levels and trends of toxaphene and chlordane-related pesticides in peregrine falcon Eggs from South Greenland. Sci Total Environ 468-469, 614-21.* |
| POPs | Eggs | *Vorkamp, K., Thomsen, M., Falk, K., Leslie, H., Møller, S., Sørensen, P.B. 2005. Temporal development of brominated flame retardants in peregrine Falcon (Falco peregrinus) eggs from South Greenland (1986–2003). Environ. Sci. Technol., 39, 8199–8206* |
| POPs | Eggs | *Vorkamp, K., Thomsen, M., Møller, S., Falk, K., Sørensen, P.B., 2009, Persistent organochlorine compounds in peregrine falcon (Falco peregrinus) eggs from South Greenland: Levels and temporal changes between 1986 and 2003. Environment International 35, 336-341.* |
| POPs | Eggs | *Wiesmüller T, Schlatterer B, Wuntke B, Schneider R. 1999. PCDDs/PCDFs, Coplanar PCBs and PCBs in Barn Owl Eggs from Different Areas in the State of Brandenburg, Germany. Bulletin of Environmental Contamination and Toxicology 63(1): 15-24.* |
| POPs | Eggs | *Wiesmüller T, Sömmer P, Volland M, Schlatterer B. 2002. PCDDs/PCDFs, PCBs, and Organochlorine Pesticides in Eggs of Eurasian Sparrowhawks (Accipiter nisus), Hobbies (Falco subbuteo), and Northern Goshawks (Accipiter gentilis) Collected in the Area of Berlin-Brandenburg, Germany. Archives of Environmental Contamination and Toxicology 42(4): 486-496.* |
| POPs | Eggs | *Baluja, G. and Hernández, L.M. 1978. Organochlorine pesticide and PCB residues in wild bird eggs from the South-West of Spain. Bull. Environ. Contam. Toxicol. 19, 655–664.* |
| POPs | Eggs | *Baum F, Conrad B (1978) Greifvögel als Indikatoren für Veränderungen der Umweltbelastung durch chlorierte Kohlenwasserstoffe. Tierärztl Umschau 12:661–668* |
| POPs | Eggs | *Weber, M., D. Schmidt and J. Hadrich. 2003. Organochlorine residues in German Osprey (Pandion haliaetus) eggs. Journal fur Ornithologie 144(1): 45-57.* |
| POPs | Eggs | *Yoccoz, N. G.; Bustnes, J. O.; Bangjord, G.; Skaare, J. U., 2009. Reproduction and survival of tawny owls in relation to persistent organic pollutants. Environment international 35, (1), 107-112.* |
| POPs | Eggs and Fat | *Kredl F, Kren K. 1986. Residual chlorinated pesticides and polychlorinated biphenyls in the Eggs and adipose tissue of wild birds.(Article in Czech). Vet Med (Praha) 31(7), 423-32.* |
| POPs | Eggs, Liver and Muscle | *Walker, C H, Hamilton, G a, Harrison, R B. 1967. Organochlorine insecticide residues in wild birds in Britain. Journal of the science of food and agricultura 18 (3): 123-129.* |
| POPs | Feathers | *Eulaers I, Jaspers VLB, Bustnes JO, Covaci A, Johnsen TV, Halley DJ, Moum T, Ims RA, Hanssen SA, Erikstad KE, Herzke D, Sonne C, Ballesteros M, Pinxten A, Eens M, 2013. Ecological and spatial factors drive intra- and interspecific variation in exposure of subarctic predatory bird nestlings to persistent organic pollutant. Environment International., 57-58, 25-33.* |
| POPs | Feathers | *Jaspers VL, Covaci A, Van den Steen E, Eens M. 2007b. Is external contamination with organic pollutants important for concentrations measured in bird feathers? Environ Int. 33(6):766-72.* |
| POPs | Feathers | *Jaspers, V.L., Voorspoels, S., Covaci, A., Lepoint, G., Eens, M., 2007a, Evaluation of the usefulness of bird feathers as a non-destructive biomonitoring tool for organic pollutants: a comparative and meta-analytical approach. Environ Int 33, 328-337.* |
| POPs | Feathers and Plasma | *Eulaers I, Jaspers VL, Halley DJ, Lepoint G, Nygård T, Pinxten R, Covaci A, Eens M. 2014b. Brominated and phosphorus flame retardants in White-tailed Eagle Haliaeetus albicilla nestlings: bioaccumulation and associations with dietary proxies (δ13C, δ15N and δ34S). Sci Total Environ. 15; 478:48-57.* |
| POPs | Feathers and Plasma | *Eulaers, I.; Covaci, A.; Herzke, D.; Eens, M.; Sonne, C.; Moum, T.; Schnug, L.; Hanssen, S. A.; Johnsen, T. V.; Bustnes, J. O.; Jaspers, V. L. B., 2011a. A first evaluation of the usefulness of feathers of nestling predatory birds for non-destructive biomonitoring of persistent organic pollutants. Environment International 37, (3), 622-630.* |
| POPs | Feathers and Preen oil | *Jaspers, V.L.B., Rodriguez, F.S., Boertmann, D., Sonne, C., Dietz, R., Rasmussen, L.M., Eens, M., Covaci, A., 2011, Body feathers as a potential new biomonitoring tool in raptors: A study on organohalogenated contaminants in different feather types and preen oil of West Greenland white-tailed eagles (Haliaeetus albicilla). Environment International 37, 1349-1356.* |
| POPs | Feathers, Liver and Muscle | *Jaspers V.L.B., Voorspoels S., Covaci A., Eens M. 2006b. Can predatory bird feathers be used as a non-destructive biomonitoring tool of organic pollutants? Biology Letters, 2, 283-285.* |
| POPs | Feathers, Liver, Muscle, Fat and Preen gland | *Eulaers I, Jaspers VL, Pinxten R, Covaci A, Eens M. 2014a. Legacy and current-use brominated flame retardants in the Barn Owl. Sci Total Environ. 15; 472:454-62.* |
| POPs | Feathers, Preen oil and Plasma | *Eulaers I, Covaci A, Hofman J, Nygård T, Halley DJ, Pinxten R, Eens M, Jaspers VL. 2011b. A comparison of non-destructive sampling strategies to assess the exposure of white-tailed eagle nestlings (Haliaeetus albicilla) to persistent organic pollutants. Sci Total Environ. 410-411:258-65. doi: 10.1016/j.scitotenv.2011.09.070. Epub 2011 Oct 20.* |
| POPs | Liver | *Crosse, J.D., Shore, R.F., Jones, K.C., Pereira, M.G. 2013. Key factors affecting liver PBDE concentrations in sparrowhawks (Accipiter nisus). Environmental Pollution 177: 171-176.* |
| POPs | Liver | *De Liguoro M1, Amorena M, Naso B, Donato A, Lucisano A. 2002. Levels of p,p'-DDE in liver of predatory birds from Calabria, Italy. Bull Environ Contam Toxicol 68(3), 377-82.* |
| POPs | Liver | *Falandysz J., Szefer P. 1983. Metals and organochlorines in a specimen of white-tailed eagle. Environmental Conservation 10, 256-258* |
| POPs | Liver | *Jaspers, V.L., Dirtu, A.C., Eens, M., Neels, H., Covaci, A., 2008, Predatory bird species show different patterns of hydroxylated polychlorinated biphenyls (HO-PCBs) and polychlorinated biphenyls (PCBs). Environ Sci Technol 42, 3465-3471.* |
| POPs | Liver | *Karlog O, Kraul I, Dalgaard-Mikkelsen S. 1971. Residues of polychlorinated biphenyls (PCB) and organochlorine insecticides in liver tissue from terrestrial Danish predatory birds. Acta Vet Scand 12(2), 310-2.* |
| POPs | Liver | *Luzardo OP, Ruiz-Suárez N, Henríquez-Hernández LA, Valerón PF, Camacho M, Zumbado M, Boada LD.2014. Assessment of the exposure to organochlorine pesticides, PCBs and PAHs in six species of predatory birds of the Canary Islands, Spain. Sci Total Environ. 2014 Feb 15;472:146-53. doi: 10.1016/j.scitotenv.2013.11.021. Epub 2013 Nov 27.* |
| POPs | Liver | *Tarhanen, J., Koistinen, J., Paasivirta, J., Vourinen, P. J., Koivusaari, J., Nuuga, I., Kannan, N., and Tatsukawa, R. 1989. Toxic significance of planar aromatic compounds in Baltic ecosystem—New studies on extremely toxic coplanar PCBs. Chemosphere 18, 1067-1077.* |
| POPs | Liver | *Van Drooge, B., Mateo, R., Vives, Í., Cardiel, I., Guitart, R., 2008, Organochlorine residue levels in livers of birds of prey from Spain: Inter-species comparison in relation with diet and migratory patterns. Environmental Pollution 153, 84-91.* |
| POPs | Liver | *Wienburg, C.L., Shore, R.F., 2004, Factors influencing liver PCB concentrations in sparrowhawks (Accipiter nisus), kestrels (Falco tinnunculus) and herons (Ardea cinerea) in Britain. Environmental Pollution 132, 41-50.* |
| POPs | Liver and Eggs | *Pereira MG, Murk AJ, Van den Berg H, Walker LA, Shore RF. 2014. How much do PCB toxic equivalents account for PHAH toxicity in predatory birds? Environ Pollut 193, 240-6.* |
| POPs | Liver and Fat | *Hela DG, Konstantinou IK, Sakellarides TM, Lambropoulou DA, Akriotis T, Albanis TA. 2006. Persistent organochlorine contaminants in liver and fat of birds of prey from Greece. Arch Environ Contam Toxicol. 50(4):603-13.* |
| POPs | Liver and Fat | *Kenntner N, Krone O, Oehme G, Heidecke D, Tataruch F. 2003a. Organochlorine contaminants in body tissue of free-ranging white-tailed eagles from northern regions of Germany. Environ Toxicol Chem. 22(7):1457-64.* |
| POPs | Liver and Muscle | *Jaspers, V.L.B., Covaci, A., Voorspoels, S., Dauwe, T., Eens, M., Schepens, P., 2006a. Brominated flame retardants and organochlorine pollutants in aquatic and terrestrial predatory birds of Belgium: levels, patterns, tissue distribution and condition factors. Environmental Pollution 139, 340-352.* |
| POPs | Liver and Muscle | *Voorspoels, S., Covaci, A., Jaspers, V.L., Neels, H., Schepens, P., 2007, Biomagnification of PBDEs in three small terrestrial food chains. Environ Sci Technol 41, 411-416.* |
| POPs | Liver and Muscle | *Licata, P., F. Naccari, G. Dugo, V. Fotia, V. Lo Turco, A. G. Potorti and G. Di Bella. 2012. Organochlorine pesticides and polychlorinated biphenyls in common buzzard (Buteo buteo) from Sicily (Italy). Environmental Monitoring and Assessment 184(5): 2881-2892.* |
| POPs | Liver, Fat and Brain | *María-Mojica P. 1996. El Cernícalo vulgar (Falco tinnunculus) como biomonitor de la contaminación ambiental por insecticidas organoclorados en la Región de Murcia. Tesina de Licenciatura, Universidad de Murcia.* |
| POPs | Liver, Fat and Brain | *María-Mojica P. 1998. Biomonitorización de la exposición a insecticidas organoclorados en aves rapaces de la Región de Murcia. Tesis Doctoral. Universidad de Murcia.* |
| POPs | Liver, Fat, Brain, Kidney | *Sierra M, Terán MT, Gallego A, Diez MJ, Santiago D. 1987. Organochlorine contamination in three species of diurnal raptors in Leon, Spain. Bull. Environ. Contam. Toxicol 38, 254–260.* |
| POPs | Liver, Fat, Brain, Kidney and Muscle | *Sierra M, Santiago D. 1987. Organochlorine pesticide levels in barn owls collected in León, Spain. Bulletin of Environmental Contamination and Toxicology 38(2): 261-265.* |
| POPs | Liver, Kidney and Muscle | *González AS, Lage Y, Simal LJ. 2002. Determination of polycyclic aromatic hydrocarbons from buzzards (Buteo buteo) and tawny owl (Strix aluco) by liquid chromatography with fluorescence detection. J AOAC Int 85(1), 141-5.* |
| POPs | Liver, Kidney and Muscle | *López y López-Leitón, T. J., Álvarez-Piñeiro, M. E., Lage-Yusty, M.A, Simal-Lozano, J. 2001. Aliphatic hydrocarbons in birds of prey from Galicia (NW Spain). Ecotoxicology and Environmental Safety 50(1): 44-47.* |
| POPs | Liver, Kidney and Muscle | *López-López TJ, Alvarez-Piñeiro ME, Lage-Yusty MA, Simal-Lozano J. 2001. PCBs in three predatory birds from Galicia (NW Spain). Bull Environ Contam Toxicol 66(4), 497-503.* |
| POPs | Liver, Muscle and Eggs | *Leslie HA, Leonards PE, Shore RF, Walker LA, Bersuder PR, Morris S, Allchin CR, Boer Jd.2011. Decabromodiphenylether and hexabromocyclododecane in wild birds from the United Kingdom, Sweden and The Netherlands: Screening and time trends. Chemosphere 82(1), 88-95.* |
| POPs | Muscle | *Ólafsdóttir, K., Petersen, A.E., Thórdardóttir, S., Jóhannesson, T. 1995. Organochlorine residues in Gyrfalcons (Falco rusticolus) in Iceland. Bull. Environ. Contam. Toxicol. 55, 382±389.* |
| POPs | Muscle and Egg | *Falandysz J1, Strandberg L, Mizera T, Kalisińska E. 2000. The contamination of white-tailed sea eagles with organochlorines in Poland.(Article in Polish). Rocz Panstw Zakl Hig 51(1):7-13.* |
| POPs | Preen oil | *Van den Brink NW, Groen NM, De Jonge J, Bosveld ATC. 2003. Ecotoxicological suitability of floodplain habitats in The Netherlands for the little owl (Athene noctua vidalli). Environmental Pollution 122(1): 127-134.* |
| POPs | Serum | *Gómara, B., Ramos, L., Gangoso, L., Donázar, J.A., González, M.J. 2004. Levels of polychlorinated biphenyls and organochlorine pesticides in serum samples of Egyptian Vulture (Neophron percnopterus) from Spain. Chemosphere 55(4): 577-583.* |
| POPs | Serum, Liver, Muscle, Brain and Fat | *Voorspoels, S., Covaci, A., Lepom, P., Jaspers, V.L., Schepens, P., 2006, Levels and distribution of polybrominated diphenyl ethers in various tissues of birds of prey. Environ Pollut 144, 218-227.* |
| POPs and Hg | Eggs | *Helander B, Olsson M, Reutergårdh L, 1982. Residue levels of organochlorine and mercury compounds in unhatched eggs and the relationships to breeding success in White-tailed Sea Eagles Haliaeetus albicilla in Sweden. Holarct. Ecol. 5:349-366.* |
| POPs and Hg | Eggs | *Henny C.J., Galushin V.M., Dudin P., Khrustov A.V., Mischenko A.L., Moseikin V.N., Sarychev V., Turchin V. 1998. Organochlorine pesticides, PCBs, and mercury in hawk, falcon, eagle and owl eggs from the Lipetsk, Voronezh, Novgorod and Saratov Regions, Russia, 1992-1993. J Raptor Res 32:143-150.* |
| POPs and Hg | Eggs | *Newton I., Dale L., Little B. 1999a. Trends in organochlorine and mercurial compounds in the eggs of British Merlind Falco columbarius. Bird Study 46(3), 356-362.* |
| POPs and Hg | Eggs | *Nygåard, T., 1999, Long Term Trends in Pollutant Levels and Shell Thickness in Eggs of Merlin in Norway, in Relation to its Migration Pattern and Numbers. Ecotoxicology 8, 23-31.* |
| POPs and Hg | Eggs | *Nygåard, T., Gjershaug, J.O., 2001, The Effects of Low Levels of Pollutants on the Reproduction of Golden Eagles in Western Norway thanks. Ecotoxicology 10, 285-290.* |
| POPs and Hg | Liver | *Amarowicz, R., Szymkiewicz, M., Glazewski, R., Korczakowska, B., Markiewicz, K., Mellin, M., 1989. Content of chlorinated hydrocarbons, polychlorinated biphenyls and heavy metals in tissue of the white tailed eagle and lesse spotted eagle. Przegl. Zool. 33, 613–617.* |
| POPs and Hg | Liver | *Falandysz J. 1984. Metals and organochlorines in a female white-tailed eagle from Uznam Island, southwestern Baltic Sea. Environmental Conservation 11, 262-263* |
| POPs and Hg | Liver | *Newton I., Wyllie I., Asher A. 1993. Long-term trends in organochlorine and mercury residues in some predatory birds in Britain. Environ Pollut 79:143-151.* |
| POPs and Hg | Liver and Eggs | *Frøslie A, Holt G, Norheim G. 1986. Mercury and persistent chlorinated hydrocarbons in owls Strigiformes and birds of prey Falconiformes collected in Norway during the period 1965-1983. Environmental Pollution Series B, Chemical and Physical 11(2): 91-108.* |
| POPs and Hg | Liver and Kidney | *Falandysz J., Jakuezun B., Mizera T. 1988. Metals and organochlorines in four female white-tailed eagles. Marine Pollution Bulletin 19:521-526.* |
| POPs, Cd and Rodenticides | Blood, Eggs and Liver | *Gómez-Ramírez, 2011. Eagle owl (Bubo bubo) as biomonitor species of environmental persistent contaminants in the Southeastern Spain. PhD, University of Murcia, Spain.* |
| POPs, Hg and Pb | Eggs | *Pain, D.J., Burneleau, G., Bavoux, C., Wyatt, C., 1999, Levels of polychlorinated biphenlyls, organochlorine pesticides, mercury and lead in relation to shell thickness in marsh harrier (Circus aeruginosus) eggs from Charente-Maritime, France. Environmental Pollution 104, 61-68.* |
| POPs, Hg, Pb and Cd | Eggs | *González LM, Hiraldo F. 1988. Organochlorine and heavy metal contamination in the Eggs of the Spanish Imperial Eagle (Aquila (heliaca) adalberti) and accompanying changes in Eggshell morphology and chemistry. Environ Pollut. 51(4), 241-58.* |
| POPs, Hg, Pb and Cd | Eggs | *Hernández, L.M., González, M.J., Rico, M.C., Fernández, M.A. and Aranda, A. 1988b. Organochlorine and heavy metal residues in falconiforme and ciconiforme eggs (Spain). Bull. Environ. Contam. Toxicol. 40, 86–93.* |
| POPs, Hg, Pb and Cd | Eggs | *Hernández, L.M., Rico, M.C., Gonzalez, M.J., Hernan, M.A., Fernandez, M.A., 1986, Presence and Time Trends of Organochlorine Pollutants and Heavy Metals in Eggs of Predatory Birds of Spain. Journal of Field Ornithology 57, 270-282.* |
| POPs, Hg, Pb and Cd | Eggs | *Negro, J.J., Donázar, J.A., Hiraldo, F., Hernández, L.M., Fernández, M.A., 1993, Organochlorine and heavy metal contamination in non-viable eggs and its relation to breeding success in a Spanish population of Lesser Kestrels (Falco naumanni). Environmental Pollution 82, 201-205.* |
| POPs, Hg, Pb and Cd | Liver and Eggs | *González, M.J., Rico, M.C., Fernández-Aceytuno, M.C., Hernández, L.M., Baluja, G. 1983. Xenobiotic contamination of the Doñana National Park. II: Residues of organochlorine insecticides, polychlorinated biphenyls and heavy metals in Falconiforme and Strigiforme birds. Doñana Acta Vertebrata 10, 177–189. (In Spanish).* |
| POPs, Hg, Pb and Cd | Liver and Kidney | *Kenntner, N., Krone, O., Altenkamp, R., Tataruch, F., 2003b, Environmental Contaminants in Liver and Kidney of Free-Ranging Northern Goshawks (Accipiter gentilis) from Three Regions of Germany. Archives of Environmental Contamination and Toxicology 45, 0128-0135.* |
| POPs, Hg, Pb and Cd | Liver and Kidney | *Krone O, Stjernberg T, Kenntner N, Tataruch F, Koivusaari J, Nuuja I. 2006. Mortality factors, helminth burden, and contaminant residues in white-tailed sea eagles (Haliaeetus albicilla) from Finland. Ambio 35 (3): 98-104.* |
| POPs, Hg, Pb and Cd | Liver and Kidney | *Krone O, Wille F, Kenntner N, Boertmann D,Tataruch F. 2004. Mortality factors, environmental contaminants, and parasites of white-tailed sea eagles from Greenland. Avian Diseases 48:417-424.* |
| POPs, Hg, Pb, Cd, Rodenticides, Pharmaceuticals | Liver | *Lemarchand, C, Rosoux, R, Pénide, ME, Berny, B. 2012. Tissue concentrations of pesticides, PCBs and metals among ospreys, Pandion haliaetus, collected in France. Bulletin of Environmental Contamination and Toxicology 88, 89–93.* |
| POPs, Pb and Cd | Blood | *Navas, I., Martínez-López, E., Gómez-Ramírez, P., Hernández-García, A., Molina, I., Romero, D., María-Mojica, P. and García- Fernández, A.J. 2005a. Heavy metals and organochlorine pesticides in raptors from Wildlife Recovery Centres of Andalusia. Rev. Toxicol. 22, 114. (In Spanish).* |
| POPs, Pb and Cd | Eggs | *Hernández, L.M., Fernández, M.A. and González, M.J. 1989. Total PCBs and PCB congeners in Spanish imperial eagle eggs. Bull. Environ. Contam. Toxicol. 43, 725–732.* |
| POPs, Pb and Cd | Eggs | *Hernández, L.M., González, M.J. and Fernández, M.A. 1988a. Organochlorines and metals in Spanish imperial eagle eggs. Environ. Conserv. 15, 363–364.* |
| POPs, Pb and Cd | Liver, Kidney, Bone and Fat | *Movalli, P., Vigorita, V., Sangiorgi, E. and Lambertini, M. 1993. Accumulation of Pb, Cd, Cr, Cu, Zn and organochlorine compounds in birds of prey tissues. Proc. Regional Meeting, Society of Ecotoxicology and Environmental Safety (SECOTOX), p.140; Ist. Sup. Sanita', 26-29 September 1993, Rome, Italy.* |
| Rodenticides | Blood | *Gómez-Ramírez P, Martínez-López E, María-Mojica P, García-Fernández AJ. 2012b. A modification of quechers method to analyse anticoagulant rodenticides using small blood samples. Revista de Toxicología 29: 10-14* |
| Rodenticides | Liver | *Christensen TK, Lassen P, Elmeros M. 2012. High exposure rates of anticoagulant rodenticides in predatory bird species in intensively managed landscapes in Denmark. Arch Environ Contam Toxicol 63(3), 437-44.* |
| Rodenticides | Liver | *Hughes J, Sharp E, Taylor MJ, Melton L, Hartley G. 2013. Monitoring agricultural rodenticide use and secondary exposure of raptors in Scotland. Ecotoxicology 22(6), 974-84.* |
| Rodenticides | Liver | *Lambert O, Pouliquen H, Larhantec M, Thorin C, L'Hostis M. 2007. Exposure of raptors and waterbirds to anticoagulant rodenticides (difenacoum, bromadiolone, coumatetralyl, coumafen, brodifacoum): epidemiological survey in Loire Atlantique (France). Bull Environ Contam Toxicol 79(1), 91-4.* |
| Rodenticides | Liver | *Langford KH, Reid M, Thomas KV. 2013. The occurrence of second generation anticoagulant rodenticides in non-target raptor species in Norway. Sci Total Environ 450-451, 205-8.* |
| Rodenticides | Liver | *López-Perea JJ, Camarero PR, Molina-López RA, Parpal L, Obón E, Solá J, Mateo R. 2015. Interspecific and geographical differences in anticoagulant rodenticide residues of predatory wildlife from the Mediterranean region of Spain. Sci Total Environ 511, 259-67.* |
| Rodenticides | Liver | *Newton I, Dale L, Finnie JK, Freestone P, Wright J, Wyatt C, Wyllie I. 1998. Wildlife and Pollution: 1997/98. Joint Nature Conservation Committee, Peterborough, UK, Annual Report. JNCC Report No. 285.* |
| Rodenticides | Liver | *Newton I, Shore RF, Wyllie I, Birks JDS, Dale L, 1999b. Empirical evidence of side-effects of rodenticides on some predatory birds and mammals. In: Coward DP and Feare CJ (cols). Advances in vertebrate pest management. Filander Verlag, Fürth, Germany. 347-367.* |
| Rodenticides | Liver | *Olea PP, Sánchez-Barbudo IS, Viñuela J, Barja I, Mateo-Tomás P, Piñeiro A, Mateo R, Purroy FJ. 2009. Lack of scientific evidence and precautionary principle in massive release of rodenticides threatens biodiversity: old lessons need new reflections. Environ Conservat 36, 1-4.* |
| Rodenticides | Liver | *Ruiz-Suárez N, Henríquez-Hernández LA, Valerón PF, Boada LD, Zumbado M, Camacho M, Almeida-González M, Luzardo OP. 2014. Assessment of anticoagulant rodenticide exposure in six raptor species from the Canary Islands (Spain). Sci Total Environ 485-486, 371-6.* |
| Rodenticides | Liver | *Sánchez-Barbudo, I.S., Camarero, P.R., Mateo, R. 2012a. Primary and secondary poisoning by anticoagulant rodenticides of non-target animals in Spain. Science of the Total Environment 420: 280–288.* |
| Rodenticides | Liver | *Walker LA, Llewellyn NR, Pereira MG, Potter ED, Molenaar FM, Sainsbury AW, Shore RF. 2010a. Anticoagulant rodenticides in predatory birds 2007 & 2008: a Predatory Bird Monitoring Scheme (PBMS) report. Centre for Ecology & Hydrology, Lancaster, UK.* |
| Rodenticides | Liver | *Walker LA, Turk A, Long SM, Wienburg CL, Best J, Shore RF. 2008b. Second generation anticoagulant rodenticides in tawny owls (Strix aluco) from Great Britain. Sci Total Environ 393, 93-98.* |
| Rodenticides | Liver | *Walker, L.A., Chaplow, J.S., Llewellyn, N.R., Pereira M.G., Potter, E.D., Sainsbury, A.W., & Shore, R.F. 2013. Anticoagulant rodenticides in predatory birds 2011: a Predatory Bird Monitoring Scheme (PBMS) report. Centre for Ecology & Hydrology, Lancaster, UK.* |
| Rodenticides | Liver | *Walker, L.A., Llewellyn, N.R., Pereira, M.G., Potter, E., Sainsbury, A.W. and Shore, R.F. 2010b Anticoagulant rodenticides in predatory birds 2009: a Predatory Bird Monitoring Scheme (PBMS) report. Centre for Ecology & Hydrology, Lancaster, UK.* |
| Rodenticides | Liver | *Walker, L.A., Shore, R.F., Turk, A., Pereira, M.G. and Best, J. 2008a The Predatory Bird Monitoring Scheme: Identifying chemical risks to top predators in Britain. Ambio 37, 466-471.* |
| Rodenticides | Liver and other organs | *Sánchez-Barbudo, I.S., Camarero, P.R., Mateo, R. 2012b. Intoxicaciones intencionadas y accidentales de fauna silvestre y doméstica en España: diferencias entre Comunidades Autónomas Revista de Toxicología 29: 20-28.* |
| Rodenticides | Pellets | *Eadsforth CV, Gray A, Harrison EG. 1996. Monitoring the Exposure of Barn Owls to Second-Generation Rodenticides in Southern Eire. Pesticide Science 47 (3), 225-233.* |
